# Spatial and Temporal Structure of Environmentally‐Acquired *Caballeronia* Symbionts of a Leaffooted Bug

**DOI:** 10.1111/mec.70467

**Published:** 2026-07-27

**Authors:** Alison Ravenscraft, Suzanne E. Kelly, David R. Haviland, Johnathan E. Adamson, Martha S. Hunter

**Affiliations:** ^1^ Department of Biology University of Texas at Arlington Arlington Texas USA; ^2^ Department of Entomology University of Arizona Tucson Arizona USA; ^3^ University of California Cooperative Extension, Kern County Bakersfield California USA

## Abstract

Animals that acquire beneficial microbial symbionts from their environment risk acquiring a sub‐optimal partner, or no partner at all. The leaffooted bug *Leptoglossus zonatus* (Coreidae) acquires its *Caballeronia* (Burkholderiaceae) bacterial symbiont from the environment, presumably from local soil. Despite large contributions to the bug's fitness, young nymphs must re‐acquire the symbiont every generation. To understand how the environmental reservoir of symbiont lineages shapes the insect's biology, we examined the role of space and time in the distribution of *Burkholderia sensu lato* (including *Caballeronia*) strains in bugs and soils. We compared samples within trees, within plots, within cities and among different cities in the Southwest USA. We also sampled *Caballeronia* in *L. zonatus* within a pomegranate orchard over 2 years. We found high *Caballeronia* diversity both in soils (32 lineages) and in bugs (29 lineages). *Caballeronia* lineages were spatially structured among soils and bugs, with fewer shared as distance between samples increased. Where a bug develops, therefore, influences the symbiont strain it acquires, consistent with a process of passive spatial turnover. Also, while some *Caballeronia* subclade frequencies in bugs approximated frequencies in soils, the Coreiodea‐associated subclade of *Caballeronia* (SBE𝛿) was enriched in bugs. There was only slight turnover of strains in insects over time, suggesting that geographic variation is much more important than temporal change. Ultimately, understanding how symbiont strains of varying local benefit are distributed in space and time will help us predict how geography and seasonality are related to host fitness in environmentally acquired symbioses.

## 1 Introduction

Bacterial associates of animals are a major diversifying force in animal evolution and influence animal health, development, defence and reproduction (Mcfall‐Ngai et al. 2013). The way in which these bacteria are acquired by their host influences the nature of the association. In terrestrial arthropods, strictly maternally transmitted bacteria are common and may spread when they provide fitness benefits to their hosts or manipulate reproduction to favour infected female hosts (Bull 1983; O'Neill et al. 1997). These inherited symbionts may persist in their hosts over thousands to millions of years (Moran et al. 2005), but bottlenecks and relaxed selection on redundant metabolic products both act to reduce symbiont genome size and functional repertoire (McCutcheon and Moran 2012).

In contrast to the relatively narrow conditions allowing maternally‐transmitted symbionts to spread, free‐living bacteria that are ingested and become resident in arthropod guts may be parasitic, commensal or beneficial. Beneficial symbionts acquired from the environment may provide a diverse array of services to hosts (Ravenscraft and Coon 2025). Especially compared to bacteria with long evolutionary histories of vertical transmission in hosts, free‐living bacteria that facultatively or transiently associate with eukaryotes have large multi‐functional genomes (Takeshita et al. 2014). These genomes encode functions that buffer or counter environmental challenges for the bacterium and/or the arthropod host, including digestion of recalcitrant nutrients, detoxification of plant secondary compounds and toxin‐based host defence (Engel and Moran 2013; Mason et al. 2014; Itoh et al. 2018; Jang and Kikuchi 2020).

Symbionts acquired from the environment may also adapt to local conditions outside of their hosts, providing aspects of that adaptive function to their hosts instantaneously after acquisition (Hehemann et al. 2010; Kikuchi et al. 2012; Ravenscraft et al. 2019). Local adaptation of bacteria to climate, toxins or nutrients in a habitat may be especially beneficial to hosts that acquire them after moving into that habitat. In one compelling example, bacteria that evolved to metabolize a pesticide common in soybean fields conferred resistance to soybean bugs ( *Riptortus pedestris* ) that acquired them, although the bugs had not had previous exposure to the pesticide (Kikuchi et al. 2012). This type of local benefit depends in part on some viscosity in movement of bacteria; if bacteria mix widely and continually, hosts may not benefit from a local response of their bacterial partners. The spatial structure of environmental bacteria that regularly partner with eukaryotes is therefore of special interest.

Spatial or temporal structuring of environmentally‐acquired bacterial associates of hosts may also be important for climatic adaptation to local conditions. Temperature tolerance in cnidarian symbionts has been shown to help corals survive warming (Hoegh‐Guldberg 1999). And in a terrestrial bug‐*Caballeronia* symbiosis related to the current study system, host outcomes varied among symbiont strains in a temperature‐dependent manner: *Caballeronia* strains that benefited the insect most at cool temperatures resulted in inferior outcomes at warm temperatures, and vice versa (Stillson et al. 2025). This suggests local climatic adaptation of free‐living bacteria in terrestrial systems could also aid associated hosts with climate extremes. Conversely, temperature sensitivity of maternally‐inherited bacterial symbionts within hosts is well documented and may limit the geographic distribution of many arthropods with obligate nutritional bacterial partners (Russell and Moran 2006; Wernegreen 2012; Doremus et al. 2019).

While the factors that might cause temporal structure in the identity of hosted symbionts are unknown, we can speculate that one or more of the following processes may affect strain diversity: spring arrival of hosts from multiple distant overwintering habitats (causing a spike in symbiont diversity early in the season), seasonal changes of temperature or precipitation that influence the environmental pool of available strains, or density‐dependent factors such as horizontal transmission of strains among hosts leading to a possible reduction in strain numbers later in the season. In contrast, a lack of temporal structure in symbiont strains could suggest that hosts mix widely or acquire symbionts predominantly from one source pool of strains that remains similar in rank abundances.

While beneficial symbioses in terrestrial arthropods may be more likely to be vertically transmitted (Russell 2019), there is at least one clade in which environmental acquisition has flourished. At least seven families of hemipteran bugs in the infraorder Pentatomomorpha participate in a beneficial relationship with the free‐living bacterium *Caballeronia*, a member of the class Betaproteobacteria and family Burkholderiaceae (Kikuchi et al. 2011a; Kuechler et al. 2016). This association is both ancient and widespread, involving thousands of insect species. In all cases studied, the relationship appears to function in a similar way (Kikuchi et al. 2011a), but much of the detailed investigations to date have centered on the soybean bug *Riptortus pedestris* in the coreoid family Alydidae (Kikuchi et al. 2007, 2012; Ohbayashi et al. 2015; Itoh et al. 2019).

In the Hemiptera (‘true bugs’) that associate with *Caballeronia*, eggs and hatchlings of the insect are aposymbiotic. The second instar nymphs ingest *Caballeronia* from the environment (Kikuchi et al. 2007). After ingestion, the bacterium travels through the midgut and through a constriction that restricts all but *Caballeronia* and a few closely related genera (Ohbayashi et al. 2015; Kinosita et al. 2018). The constricted region then closes and the bacteria colonize the distal midgut 4 (M4) region, which then serves as a symbiotic organ (Fronk and Sachs 2022). A comparative transcriptome analysis of bug‐associated versus in vitro *Caballeronia* suggested that in *R. pedestris*, benefits provided by the symbiont include nitrogen from recycled metabolic wastes, amino acids and B‐vitamins (Ohbayashi et al. 2019).

Our past work in the leaffooted bug *Leptoglossus zonatus* (Coreidae) suggests that failure to acquire the symbiont in this insect results in severe consequences (Hunter et al. 2022). Only about 1/3 of nymphs reared without the symbiont survive to adulthood, and those that do take about 40% longer to develop and are approximately half the size of symbiotic adults at eclosion (Hunter et al. 2022). Lastly, aposymbiotic bugs do not reproduce (Umanzor et al. 2025).

While the location of the symbiont at the time of ingestion is not known for all systems, the ultimate reservoir of *Caballeronia* strains is the soil, where these bacteria are relatively common (Itoh et al. 2014). As a result, insects can associate with many different strains and species of *Caballeronia* (Garcia et al. 2014; Ravenscraft et al. 2020; Ohbayashi et al. 2022; Stoy et al. 2023). While it is critical for bugs to acquire some strain of *Caballeronia*, not all strains are equally beneficial, even under benign laboratory conditions (Hunter et al. 2022). Under more stressful field conditions symbiont lineages may have even more variable consequences for the bugs that acquire them, e.g., if some strains are pesticide‐degraders (Tago et al. 2015) or if conferred outcomes depend on local climate conditions (Ohbayashi et al. 2015; Stillson et al. 2025). Understanding how the symbiont community varies over space and time will therefore help clarify the consequences of environmental acquisition for host fitness.

Although *Caballeronia* is the native symbiont, some closely related bacteria can also opportunistically colonize the M4 organ. *Caballeronia* belongs to the *Burkholderia sensu lato* clade, which also includes *Burkholderia sensu stricto*—which consists primarily of animal pathogens and does not colonize the M4—and *Paraburkholderia*, a genus of predominantly plant‐associated beneficials, some of which can colonize the M4 (Itoh et al. 2019). In fact, one subclade within *Paraburkholderia*, the ‘insect‐ and plant‐ associated beneficial and environmental’ (iPBE) group, is the native symbiont of bordered plant bugs (Pyrrhocoroidea: Largidae) (Takeshita et al. 2015). The outgroup genera *Pandoraea* and *Cupriavidus* can also colonize. *Caballeronia* outcompetes these genera when they co‐colonize the M4, but in its absence, these non‐native symbionts can partially or largely rescue the fitness consequences incurred by aposymbiotic insects (Itoh et al. 2019).

In this study, we examined the spatial and temporal structure of *Caballeronia* symbionts of *L. zonatus*. This insect is common in the western United States, where it feeds on several native and exotic plant species (Ingels and Haviland 2014) and is an important pest of pomegranates, almonds and pistachios (Ingels and Haviland 2014). We tested the following hypotheses: (1) The soil reservoir of *Caballeronia* will be spatially linked to the pool of symbionts within bugs, such that bugs are more likely to share *Caballeronia* strains with nearby soil than distant soil. (2) Diversity of strains in insects will also be spatially structured, with a given bug more likely to share strains with nearby bugs than distant bugs. (3) Within a single pomegranate orchard, as time increases between bug sampling dates, bugs are less likely to share the same strains.

## 2 Methods

### 2.1 Sample Collection and DNA Extraction

Insects and soils for the spatial survey were collected in the USA in Bakersfield, CA, Fresno, CA and Tucson, AZ in September 2018. To control for potential effects of host plant species, we characterized *Caballeronia* strains in insects and soils exclusively from pomegranate trees. We sampled trees in four orchards, four unmaintained hedges and two nurseries. In each city, we collected wild bugs from three or four pomegranate sites spaced approximately 15 km apart. Within each site, we collected about eight insects from each of three trees spaced 3–30 m apart. We also collected one soil sample under each tree. Our past work characterizing *Caballeronia* (=*Burkholderia*) strain variation within the stilt bug *Jalysus wickhami* showed that a distance of 5 m can result in significant symbiont turnover (Ravenscraft et al. 2020). In total, we collected 27 soil samples and 241 *L. zonatus* bugs (90 adults, 87 fifth instar nymphs, 41 fourth instar nymphs, 10 third instar nymphs and 13 second instar nymphs).

To assess potential seasonal turnover of *Caballeronia* strain diversity, we selected a single *L. zonatus* population at a research pomegranate orchard at the University of Arizona's Veterinary Diagnostic Clinic site (Tucson, AZ, USA) and collected insects every 1.5 months from April through October (skipping sampling in winter, when bugs are dormant). Sampling took place for 2 years, starting with the spatial survey insects sampled in September 2018 and ending in October 2020. The sampling interval was chosen to census the population approximately once every one to two generations. At each visit we collected about 14 individuals (range 11–24). Over all 11 visits, we captured 65 adults, 49 fifth instars, 26 fourth instars, 19 third instars and 10 second instars, for a total of 169 insects (the first 24 of which were also included in the spatial survey).

Insects were preserved in 95% ethanol. DNA was extracted from either whole insect bodies or from dissected M4 midgut regions that house the symbiont. In both cases, bug tissue was homogenized with a microcentrifuge tube pestle, digested with proteinase K and extracted with the DNeasy Blood and Tissue Kit (Qiagen, Germantown, MD). The Powersoil DNA Isolation kit was used to extract DNA from each 0.25 g soil sample. We also extracted 10 empty tubes interspersed throughout the sample extractions. These were treated identically to the samples, including all downstream lab work and sequencing. These blank extractions allowed us to assess potential contamination from the laboratory environment or reagents.

### 2.2 Illumina Library Preparation and Sequencing

Insect and soil genomic DNA was amplified with the primers Bf (5′‐TAGCCCTGCGAAAGCCG‐3′) and Br (5′‐GCCAGTCACCAATGCAG‐3′), which amplify the *Burkholderia sensu lato* clade that includes *Caballeronia*, *Paraburkholderia* and *Burkholderia sensu stricto* (Tago et al. 2014). Each primer included an overhang adapter to enable downstream addition of sample indexes (as per Illumina's 16S Metagenomic Sequencing Library Preparation guide). Samples from the spatial survey were amplified in a volume of 20 μL with final concentrations of 0.25 μM forward primer, 0.25 μM reverse primer, 0.2 mM dNTPs, 1× Phusion HF buffer (Thermo Scientific) and 0.02 U/μL Phusion Hot Start II DNA polymerase (Thermo Scientific). The thermocycler program was: Denaturation at 98 ***°*** C for 1.5 min followed by 30 cycles of denaturation at 98 ***°*** C for 10 s, primer annealing at 61 ***°*** C for 20 s and extension at 72 ***°*** C for 50 s, with a final extension of 72 ***°*** C for 5 min. We cleaned the PCR products with magnetic beads (Rohland and Reich 2012). To differentiate the amplicons from each sample, a second short PCR was used to attach a unique pair of 8‐nucleotide barcodes to the forward and reverse ends of each amplicon (Hamady et al. 2008). The volume of this second PCR was also 20 μL. We amplified 2.5 μL of the cleaned PCR product with 0.2 μM of each barcoded primer, 0.2 mM dNTPs, 1× Phusion HF buffer and 0.02 U/μL Phusion Hot Start II DNA polymerase. Thermocycler conditions were: denaturation at 98 ***°*** C for 1.5 min followed by 8 cycles of denaturation at 98 ***°*** C for 10 s plus combined primer annealing and extension at 72 ***°*** C for 40 s, followed by a final extension of 72 ***°*** C for 5 min. PCR products were cleaned with magnetic beads, quantified fluorometrically (Qubit DS DNA HS assay, Qiagen) and an equal mass of each sample was bidirectionally sequenced on a 600 cycle paired‐end run on an Illumina Mi‐Seq platform at the University of Arizona Genetics Core.

Samples from the temporal survey were prepared using the same methods, except that we used Q5 master mix and polymerase (New England Biolabs) for the PCRs. The recipe for the first PCR was 0.25 μM forward primer, 0.25 μM reverse primer and 1× Q5 master mix and thermocycler settings were: denaturation at 98°C for 1 min, 30 cycles of denaturation at 98°C for 10 s, annealing at 50°C for 20 s and extension at 72°C for 30 s with a final extension at 72°C for 2 min. The second PCR used 0.5 μM of each barcoded primer and 8 cycles of the same thermocycler conditions, except that the annealing temperature was raised to 55°C. The library was sequenced at the University of Texas at Arlington's Life Science Core Facility.

### 2.3 Sequence Data Processing

Priming sites and poor‐quality bases were removed from the 5′ and 3′ ends of the sequences using the program cutadapt (Martin 2011). We discarded all reads that contained any unassigned bases (Ns) or had an expected error score greater than 2. We truncated the remaining reads at the first instance of a quality score less than 2. For each run, we used the DADA2 R package to merge paired ends and infer the bacterial strains present (Callahan et al. 2016). DADA2 corrects sequencing errors to infer true biological sequences using the run‐specific error rate, quality scores and number of times each sequence is observed. Data from the two runs were then merged by sequence using the DADA2 package's *mergeSequenceTables* function. We performed de novo chimera checking and removal on the combined data and assigned taxonomy using the RDP classifier and the SILVA nr99 v138 training set (Quast et al. 2013; Wang et al. 2007). Inspection of the blanks did not indicate the presence of any contaminants. Finally, the data were rarefied to 2179 sequences per sample to control for among‐sample differences in sequencing depth. Visual inspection of rarefaction curves indicated that observed diversity had plateaued in the samples by this depth. We also performed analyses on just the *Caballeronia* genus. For these analyses, we first subset the raw data to amplicon sequence variants identified as *Caballeronia*, then rarefied to the same depth of 2179 sequences per sample.

### 2.4 Phylogeny

To construct a reference phylogeny of the *Burkholderia sensu lato*, we selected all 39 of the *Caballeronia* genomes available in the Integrated Microbial Genomes (IMG) database as of June 2023, as well as a representative subset of *Paraburkholderia* (41 genomes), *Burkholderia sensu stricto* (17), *Trinickia* (4), *Cupriavidus* (3) *Pandoraea* (2) and *Ralstonia* (1) genomes (Chen et al. 2023).

We also sequenced the genomes of an additional 20 *Caballeronia* symbionts, including 19 isolated from the insects in this study (Table S1). Briefly, insects were dissected aseptically and their M4 organs were removed. A small piece of the M4 was incubated on a shaker at ambient temperature in yeast‐glucose (YG) broth or Ashdown's broth. This organ culture step improves the recovery of the symbiont (Xu et al. 2016). After 24 h, the M4 pieces were removed from the broth, homogenized with a sterile test tube pestle and plated on YG or Ashdown's agar. After 3–9 days, the resulting bacterial colonies were screened using diagnostic PCR with *Caballeronia*‐specific primers Burk 16SF‐16SR (Kikuchi et al. 2005). Confirmed *Caballeronia* isolates were frozen at −80 C in glycerol. To obtain DNA for genome sequencing, a single colony was incubated overnight in YG broth. DNA was extracted from one millilitre of the resulting culture using the Wizard Genomic DNA Purification Kit (Promega A1120, Madison, WI) and sequenced by SNPsaurus (Eugene, OR) or Plasmidsaurus (Eugene, OR) (See Table S1 for details and accession numbers).

From each of the resulting 133 genomes, we used IMG to extract five full‐length genes: *rpoB*, *rpoC*, *rplA*, *recA* and 16S rRNA. We selected the first three genes because they are the single‐copy genes shown to have the highest concordance to bacterial species phylogenies (Hassler et al. 2022). We selected *recA* because it is commonly used for identification of *Burkholderia sensu lato* species and clades. We included 16S rRNA to enable us to align our 16S rRNA amplicon sequence variants to the reference sequences. We added 34 near full‐length 16S rRNA sequences from our own *Caballeronia* isolate library (including 32 isolates from the insects in this study; Table S1) and 21 additional 16S rRNA sequences from GenBank which belonged to known symbiotic subclades (9 SBEα, 4 SBEβ, 5 SBE𝛿 and 3 iPBE (Ohbayashi et al. 2022)).

We performed protein alignments on the four protein‐coding genes and a nucleotide alignment of the 16S using MAFFT (Katoh and Standley 2013). We built a maximum‐likelihood tree on the concatenated alignments using RAxML with the GTR + Gamma model of nucleotide substitution (Stamatakis 2014), with separate partitions for each coding position of each protein coding gene plus one partition for the 16S rRNA. The reference tree's node support values were calculated using rapid bootstrapping which was halted automatically based on the MRE criterion. The tree was rooted with the outgroups *Pandoraea pulmonicola* and *Pandoraea oxalativorans* . We placed the Illumina amplicon sequence variants onto the resulting phylogeny using SEPP implemented in QIIME2 (Mirarab et al. 2012; Bolyen et al. 2019). Finally, we used the *tip_glom* function from the phyloseq R package to agglomerate sequence variants into lineages with a cophenetic distance of 0.1 (McMurdie and Holmes 2013). Grouping a phylogeny at a cophenetic distance of 0.1 means merging tips that are separated by less than 0.1 substitutions per site into a single tip; for the *Caballeronia* in this study, this appeared to equate very roughly to the species level. Sequence variants from this study were 464 or 466 base pairs in length. Concatenated reference sequences varied from 10,122 to 11,689 bp in length and the additional 16S rRNA sequences ranged from 1313 to 1456 bp. To facilitate identification of *Caballeronia* subclades in future studies, the *Burkholderia s. l*. reference phylogeny and the QIIME script for sequence placement are available in our Dryad repository.

**FIGURE 1:**
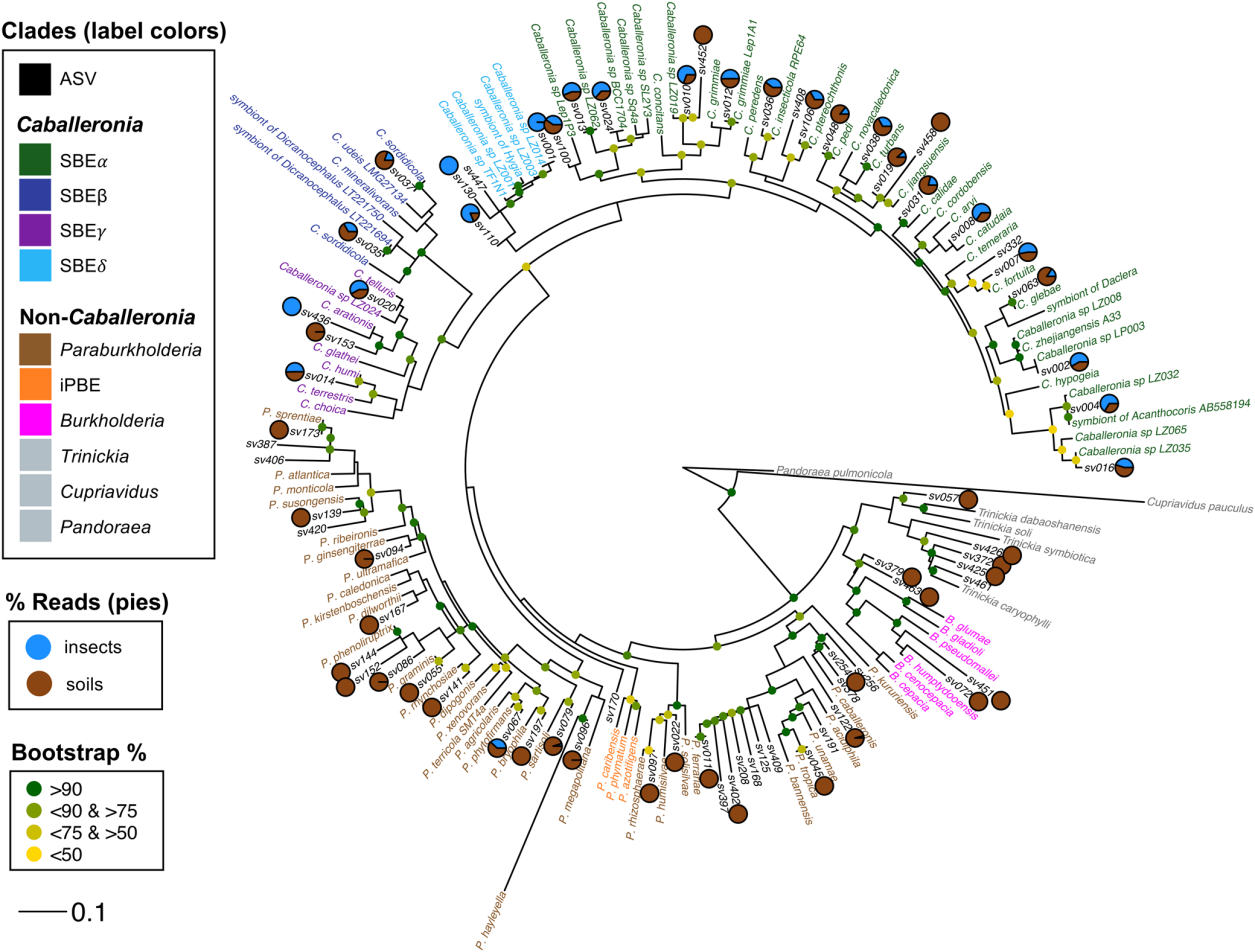
Phylogenetic placement of the most abundant *Burkholderia s. l*. lineages associated with *Leptoglossus zonatus* and local soils. A maximum‐likelihood phylogeny was built from five full‐length genes (rpoB, rpoC, rplA, recA and 16S rRNA) extracted from Burkholderiaceae reference genomes, supplemented with additional 16S rRNA sequences. Scale bar indicates 0.1 nucleotide substitutions per site. Lineages detected in this study (names starting ‘sv’) were then placed on the phylogeny with SEPP, and therefore have no bootstrap support values. Only lineages accounting for at least 1% of the reads in at least 2 samples are shown here; an equivalent phylogeny with all lineages is provided in Figure S1. Labels of the reference genomes are coloured according to their clade membership in several named groups as proposed by Ohbayashi et al. (2022). Pie diagrams indicate the normalized percentage of reads for each sequence variant detected in insects (blue) versus soil (brown). See Methods for details. Reference sequences that did not have closely related SV lineages were dropped from the figure; Figure S1 includes all reference sequences.

For each lineage, we calculated the normalized percentage of reads detected in insects versus soils by dividing the lineage's proportional abundance within the pool of all insect reads by the sum of this number plus the lineage's proportional abundance within the pool of all soil reads (see pies in Figure 1). This calculation weights the data from insects and soils equally, such that a lineage which was detected at the same proportional abundance in both insects and soils would have a normalized percentage of 50% insect reads (and equivalently, 50% soil reads).

## 3 Results

### 3.1 Diversity Patterns in Bugs and Soils

**FIGURE 2:**
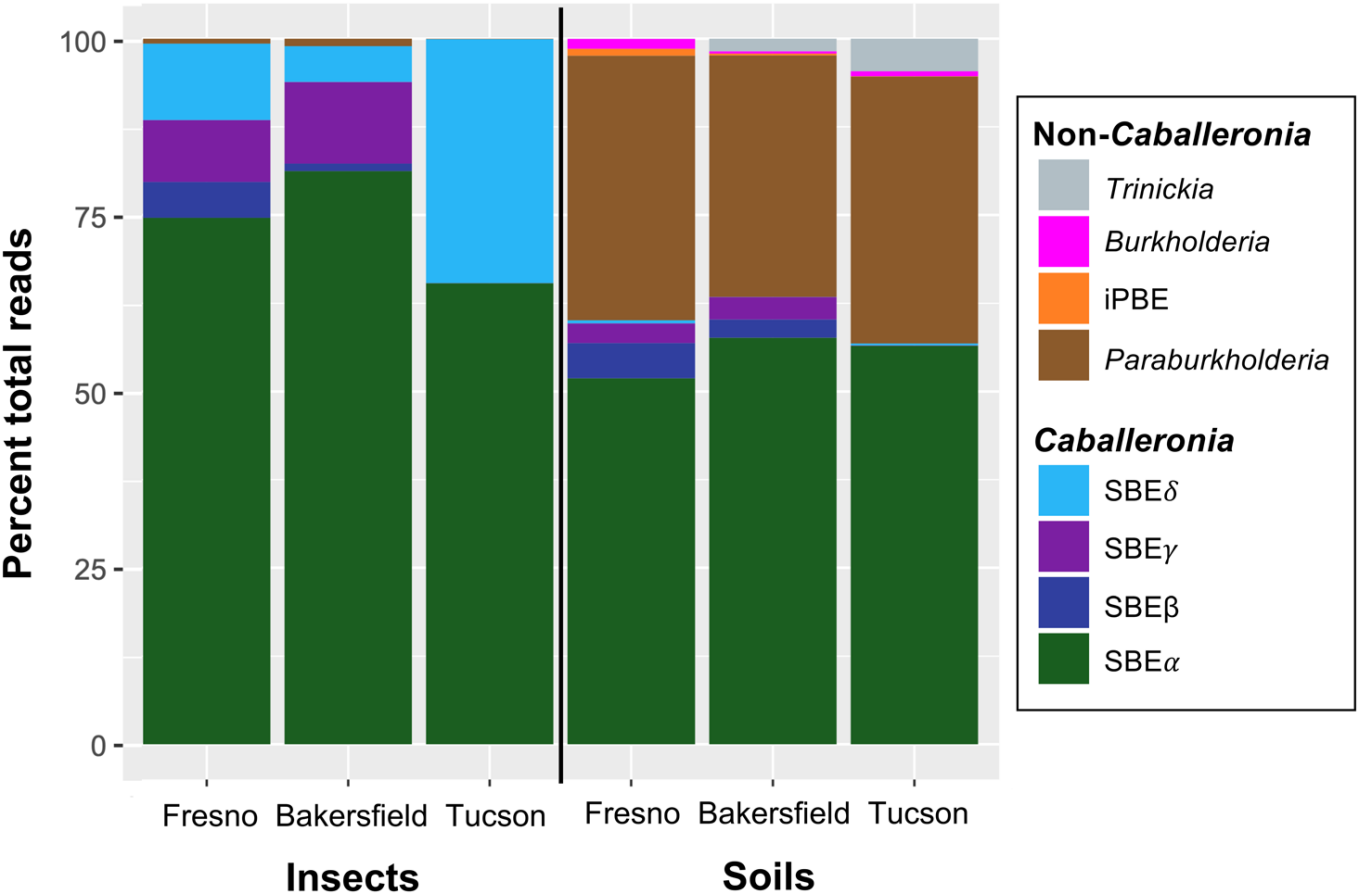
Percentage of total reads belonging to each bacterial clade from bugs and soils in Fresno, Bakersfield and Tucson. Bug percentages from Tucson include insects sampled from one pomegranate orchard over the course of 2 years. An equivalent chart for just *Caballeronia* is available in Figure S2.

**FIGURE 3:**
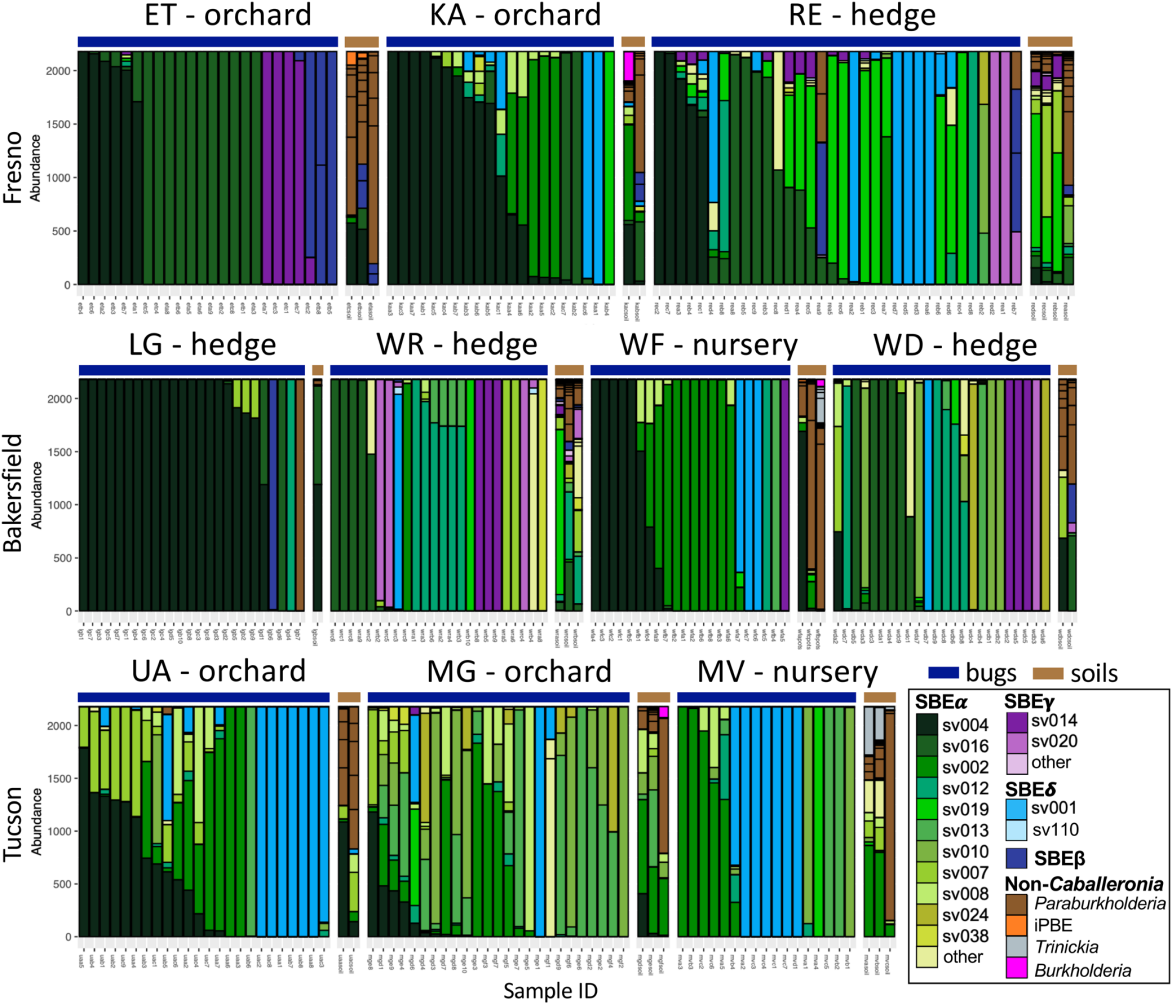
Relative abundances of *Burkholderia s. l*. lineages in populations of *Leptoglossus zonatus* and associated soils in the western USA. Each bar represents an individual insect or soil sample. All samples were rarefied to 2179 reads. Colours indicate abundances of the most common lineages in bugs (defined as those strains that accounted for 5% or more of the sequences in at least 5 bugs; rarer lineages were assigned a single colour per clade: Light green for SBEα, dark blue for SBEβ and light purple for SBE𝛾). Samples are grouped by sample type (bug or soil) and site. Samples within a site are sorted according to the abundances of the lineages, with the most abundant sorted first, followed by the second most abundant, etc. Sites are arranged from west to east in each city.

Our quality‐filtered dataset comprised 10,116,305 *Burkholderia sensu lato* reads from bugs and 512,772 *Burkholderia s. l*. reads from soil samples. After rarefaction and clustering of closely related sequence variants, we detected a total of 37 *Burkholderia s. l*. lineages in bugs and 109 in soils (Figure 1; Figure S1). Bugs hosted *Caballeronia* almost exclusively, whereas soils were dominated by a mixture of *Caballeronia* and *Paraburkholderia*, a genus often associated with plants (Figures 2 and 3).

Within the native symbiotic genus *Caballeronia*, we detected 29 lineages in bugs and 32 lineages in soils, of which 24 were shared between bugs and soils. Individual bugs hosted a median of 2 *Caballeronia* symbiont lineages (interquartile range (IQR) 1–4, max = 11) and individual soil samples a median of 9 (IQR 5–13, max = 19). It was not uncommon for multiple lineages of *Caballeronia* to co‐occupy an insect (Figure 3). When a bug was occupied by multiple lineages, one tended to be substantially more abundant than the others. Three lineages accounted for almost two thirds of all sequences from bugs: SV001 with 22% of total bug reads, SV004 with 21% and SV002 with 15%.

The relative abundances of *Caballeronia* subclades in bugs differed between cities (Figure 2; Figure S2). Despite the fact that *Leptoglossus* is a member of the Coreidae, the SBEα clade was much more abundant in *L. zonatus* in the current study (accounting for 65%–81% of total insect sequences among cities) than the Coreoidea‐associated SBE𝛿 clade. SBE𝛿 was the second most abundant in Fresno (where it comprised 11% of total insect sequences), while SBE𝛾 was the second most common in Bakersfield (12% of insect sequences). SBE𝛿 was markedly more abundant in Tuscon (35% of insect sequences). Notably, SBE𝛿 was far more abundant in insect populations than it was in their local soils,where it accounted for only 0.02%–0.4% of total reads, while in contrast, the abundances of the SBEα, SBEβ and SBE𝛾 clades were broadly similar between insects and soils.

*Caballeronia* communities differed significantly among site types (orchards, hedges and nurseries) in both bugs and soils when site type was included as the sole predictor (bug PERMANOVA: *R* ^2^ = 0.04, *p* = 0.001; soil PERMANOVA: *R* ^2^ = 0.25, *p* = 0.004). However this result appeared to be driven by location, since there were no consistent differences apparent among site types (Figure 3; Figure S3). A few lineages (3 in bugs and 5 in soils) differed significantly in relative abundance across site types (DESeq2, all *p* < 0.01), but these differences did not appear meaningful upon visual comparison (Figure S4).

### 3.2 Spatial Patterns

**FIGURE 4:**
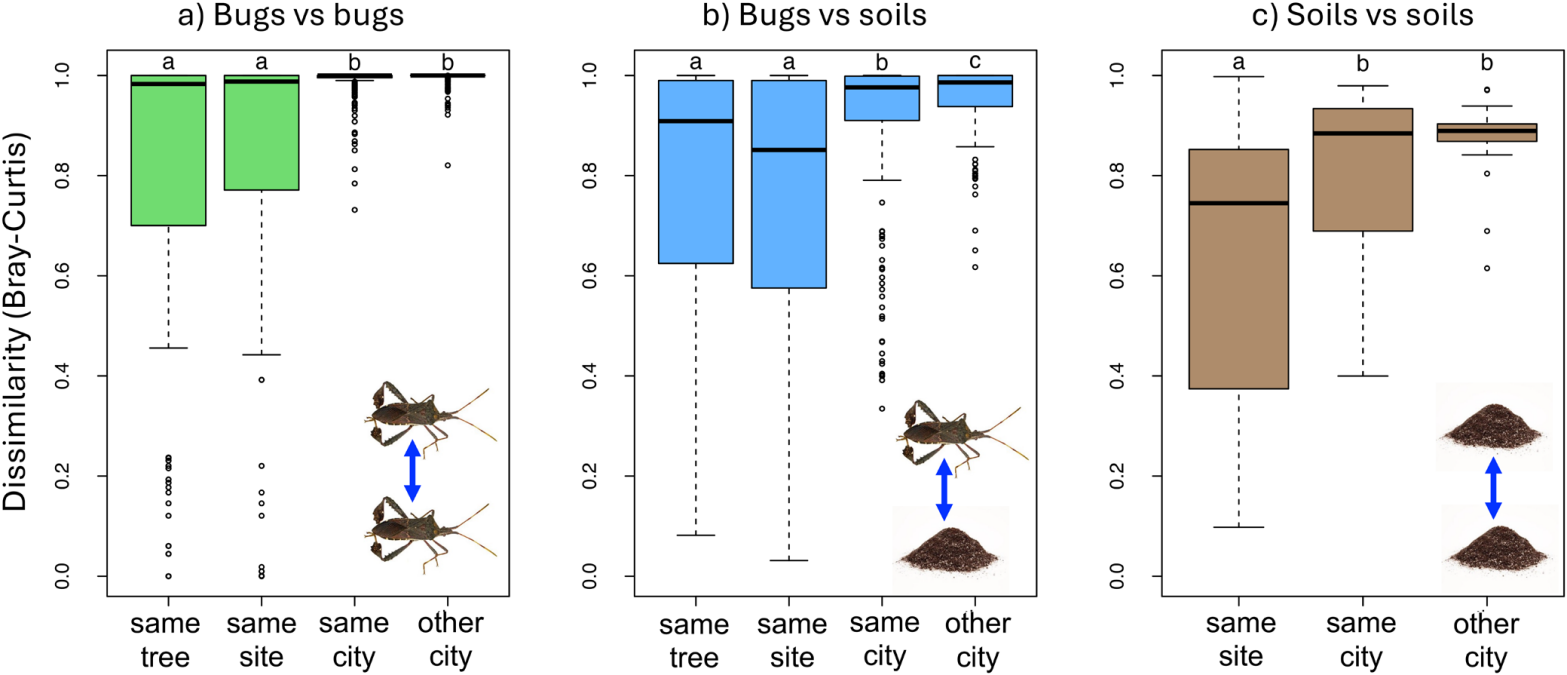
Bray–Curtis dissimilarity between samples at different spatial scales. Every point represents the Bray–Curtis dissimilarity in *Caballeronia* lineage composition between (a) a pair of bugs, (b) a bug and a soil sample or (c) a pair of soil samples. Pairwise dissimilarities are grouped according to whether the samples came from the same tree, different trees in the same site, different sites in the same city or different cities. Box plots depict medians and interquartile ranges of the data. Whiskers are placed at 1.5 times the interquartile range or, if all data fall within this range, they are placed at the most extreme value measured. Letters above the box plots indicate statistically significant differences at *p* < 0.05. The Bray–Curtis metric ranges from a score of 0, which indicates that two samples share identical lineages at identical relative abundances, to a score of 1, indicating that two samples don't share any lineage in common.

There was a lot of variation among bugs and among soils in the identities and abundances of *Caballeronia* lineages hosted (Figure 3). However, beta diversity analysis (Bray‐Curtis) indicated that differences increased with increasing distance between pairs of samples (bug vs. bug, bug vs. soil and soil vs. soil; Figure 4). Bugs tended to share more symbiont lineages at more similar abundances with individuals from the same tree and the same site than with individuals from different sites. Results were similar when we analysed only data from orchard sites, only hedge sites, or orchard and hedge sites (excluding nurseries) (Figure S5). Sites within cities were separated by a median distance of 16 km (IQR 8–31 km), indicating that there is a marked increase in turnover of *Caballeronia* strains at the scale of tens of kilometres. Similarly, PERMANOVA analysis indicated that the composition of bug‐hosted *Caballeronia* differed among states, cities and sites but not among trees within a site and free‐living soil *Caballeronia* differed among states (Tables 1 and 2). Differences between sites accounted for the greatest percent variation in bug‐hosted symbiont communities (16%). Results were qualitatively similar when the same analyses were performed on all *Burkholderia s. l*.

**TABLE 1:** *Caballeronia* lineages in bugs—PERMANOVA results.

	df	Sum of squares	*R* ^2^	*F*	Pr (> *F*)
State	1	4.6	0.045	13.3	0.001***
City	1	1.3	0.013	3.8	0.002**
Site	7	16.1	0.160	6.7	0.001***
Tree	5	2.3	0.023	1.3	0.070
Residuals	224	76.9	0.760		
Total	238	101.1	1.000		

*Note:* ** indicates *p* ≤ 0.01, *** indicates *p* ≤ 0.001.

**TABLE 2:** *Caballeronia* lineages in soils—PERMANOVA results.

	df	Sum of squares	*R* ^2^	*F*	Pr (> *F*)
State	1	1.1	0.171	3.5	0.007**
City	1	0.2	0.023	0.5	0.812
Site	7	2.8	0.412	1.2	0.28
Tree	5	0.7	0.100	0.4	0.99
Residuals	6	2.0	0.294		
Total	20	6.7	1.000		

** indicates *p* ≤ 0.01.

Finally, to estimate the upper bound on how much of an insect's *Caballeronia* could have originated from soils at different spatial scales, we quantified the percentage of *Caballeronia* reads in each insect (rarefied data) that belonged to *Caballeronia* lineages which were also detected in soils (unrarefied data). A median of 98.7% (IQR 0.6%–100%, minimum 0%) of *Caballeronia* reads in an insect belonged to lineages which were also detected in soil collected from the same tree, 100% (IQR 88.5%–100%, minimum 0%) in soils collected from the same site, 100% (IQR 100%–100%, minimum 0%) in soils collected from the same city and 100% (IQR 100%–100%, minimum 67.1%) in all collected soils. Younger insects tended to be more likely than older insects to host lineages present in soils from the same site, though this trend was only marginally significant (quasibinomial linear model predicting percentage of insect reads shared with patch soils: coefficient = −0.3, df = 1, scaled dev = 2.9, *p* = 0.08).

### 3.3 Temporal Patterns

**FIGURE 5:**
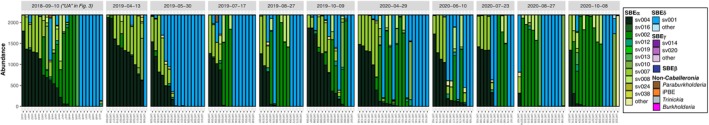
Relative abundances of *Burkholderia s. l*. lineages in *Leptoglossus zonatus* from the University of Arizona pomegranate orchard over time. Each bar represents an individual insect. Samples are grouped by visit date. All samples were rarefied to 2179 reads. Samples within a visit are sorted according to the abundances of the lineages, with the most abundant sorted first, followed by the second most abundant and so on (The first panel is the same as the UA panel in Figure 3).

**FIGURE 6:**
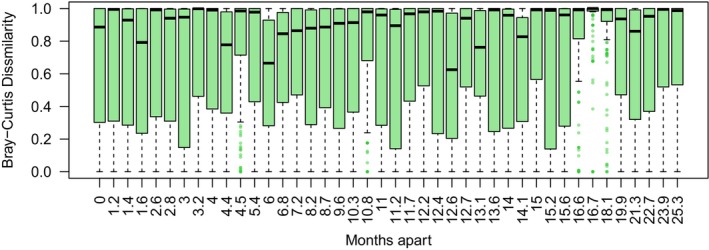
Bray–Curtis dissimilarity vs. months between sample collections in the Tucson pomegranate orchard. Every point represents the Bray–Curtis dissimilarity in *Caballeronia* lineage composition between a pair of insects plotted against the number of months elapsed between the collection dates of those insects. Box features are as in Figure 4. The lack of a systematic shift in dissimilarity over time indicates the absence of directional changes in symbiont lineage. If there was a directional shift in lineages over time, the Bray–Curtis dissimilarity would increase from left to right.

There was also significant variation in the lineages hosted by bugs at a single site over time (Figure 5). The composition of hosted *Caballeronia* lineages varied among timepoints (PERMANOVA: df = 1, *F* = 3.2, *p* = 0.03), but the degree of difference in hosted symbiont diversity increased only slightly as the time between samples increased (a quasibinomial generalized linear model predicted an increase of 0.01 in Bray–Curtis dissimilarity per month, df = 1, *p* < 0.001; Figure 6). In other words, the *Caballeronia* lineages hosted by bugs at one site underwent only a small directional shift over 2 years, or approximately 10 bug generations. The *Caballeronia* in two bugs collected a month and a half apart were thus slightly more similar to each other, on average, than two bugs collected 2 years apart.

## 4 Discussion

We characterized *Burkholderia s. l*. strains in stands of pomegranate in both the leaffooted bug *L. zonatus* and in soils underneath the trees at sites in Arizona and California. As spatial distance increased, differences in *Caballeronia* composition increased among bugs, among soils and between bugs and soils (Figure 4). We found a marked increase in spatial turnover of *Caballeronia* strains at the scale of tens of kilometres. These results support our first two hypotheses, specifically that bugs are more likely to share *Caballeronia* strains with nearby soil than distant soil and that an individual bug is more likely to share *Caballeronia* strains with nearby bugs than distant bugs. However, our third hypothesis received weaker support: dissimilarity between bugs increased only slightly with more time between samples. This suggests that location, much more than time, predictably determines the symbiont strain acquired by these insects.

### 4.1 Spatial Patterns

Ultimately, the finding that *Caballeronia* strain composition in soils turns over at approximately the same spatial scale as it does in insects suggests that nymphs are limited by which microbial partners are available in their local environment. So, contrary to the Baas‐Becking assumption, everything (or every potential symbiont) is not everywhere for these bugs (Baas‐Becking 1934). Because different symbiont strains may have different fitness outcomes for the bugs (Hunter et al. 2022; Stillson et al. 2025) and confer different adaptive benefits, such as pesticide resistance or temperature tolerance, our findings raise the possibility that symbiont‐conferred benefits or liabilities may be geographically delineated in wild *L. zonatus* populations. An average *L. zonatus* in Sacramento may therefore not have the same fitness as one in Tucson, despite similar genetic backgrounds of the bugs themselves (Joyce et al. 2017).

The increased overlap of *Caballeronia* strains between soils and bugs at smaller spatial scales is not surprising. Ultimately, bugs must be acquiring the symbiont from replicating populations in the soil, whether the proximal site of acquisition is the soil or nearby on tree foliage. It is less clear, however, whether symbionts move exclusively from soil to *L. zonatus* bugs or whether there is some horizontal transmission among bugs. Although strict environmental acquisition appears to be the rule in *R. pedestris* (Ohbayashi et al. 2015), adult squash bugs (Coreidae) and stilt bugs (Berytidae) can indirectly provide the symbiont to nymphs via inoculation of their local environment (Ravenscraft et al. 2020; Acevedo et al. 2021). Recent research on squash bugs and preliminary data in *L. zonatus* shows that *Caballeronia* is excreted in frass and can be acquired by nymphs confined with frass (Villa et al. 2023; Sullivan et al. unpubl. data). *Leptoglossus phyllopus*, a congener of *L. zonatus*, can enrich the *Caballeronia* population in the soil underneath it (Parajuli et al. 2026). These results suggest that *Caballeronia* in the soil under pomegranates may be subsidized by excretion of *L. zonatus* , leaving the net direction of *Caballeronia* strain movement between habitats (soil to bug or bug to soil) unclear and raising the possibility that the spatial scale of turnover in *Caballeronia* strains is determined as much by the dispersal distance of *L. zonatus* as by passive movement of soil bacteria via wind and water (Yang and Van Elsas 2018).

The increased tendency for younger insects to be colonized by local soil lineages (those from the same site), while only marginally significant, makes sense for an environmentally acquired symbiosis with a narrow developmental window of acquisition. In *R. pedestris* and likely *L. zonatus* , nymphs must acquire *Caballeronia* during a specific developmental period, ideally in the early second instar (Kikuchi et al. 2011b). After a symbiont has colonized the M4 organ, the constricted region of the gut seals shut, preventing entry of later colonists. The *Caballeronia* strains an insect possesses, therefore, should be derived from the location where the individual lived during its second instar. Adults can fly; older individuals are therefore more likely to have moved to different trees and sites after acquisition of their symbiont.

### 4.2 Temporal Patterns

While we found strong spatial structuring of *Caballeronia* strains, temporal structuring was comparatively weak. Sampling of symbiont strains over a two‐year period in one orchard showed variation in the lineages hosted by bugs at this single site over time (Figure 5). However, directional change in strain composition was minimal over the entire 2‐year period. This means a bug sampled 6 weeks after another one was only slightly less likely to have a different strain than one sampled a year later. These results confirm our third hypothesis, that bugs are less likely to share the same strains with increasing time, but show that *Caballeronia* communities respond more strongly to space than time. Our results suggest no clear response of *Caballeronia* strain diversity in bugs to seasonal temperature differences, as one might expect if there were large differences in performance of bugs with particular strains during the summer heat. Nor do the results indicate any large increase in strain differences early in the season when immigration to the orchard may occur. Instead, the temporal fluctuations we observed in the insect population might potentially have been due to relatively small, stochastic changes in the relative abundances of different *Caballeronia* lineages in the local soil. A caveat, however, is that this analysis is not sensitive to small differences and would not necessarily detect a low‐abundance strain dropping out, for example due to poor performance of bugs with a particular strain under temperature stress, nor the arrival of a few new, rare strains in bugs from other areas.

### 4.3 Comparison to Diversity in Another *Caballeronia*‐Hosting Bug Species

The number of lineages of *Caballeronia* revealed in samples of *L. zonatus* in California and Arizona (29) is roughly equivalent to the *Caballeronia* diversity found in the stilt bug *Jalysus wickhami* (Berytidae). In *J. wickhami* we found 61 sequence variants across the USA (Ravenscraft et al. 2020). These SVs clustered to 24 *Caballeronia* lineages at the cophenetic distance of 0.1, which we used to group ASVs into lineages in the current study and which equates roughly to the level of *Caballeronia* species. In both insects, there appears to be a variety of possible lineages with which bugs may associate, even at particular sites. A clear difference in symbiont diversity patterns between the two species, however, is the skew in strain dominance. In *J. wickhami* the most abundant lineage accounted for 75% of the reads, functionally reducing the diversity of associates (Ravenscraft et al. 2020). In the current study in *L. zonatus*, the most abundant lineages represent 22% (SV001), 21% (SV004) and 15% (SV002) of total reads derived from bugs. It is not clear yet what drives this difference in evenness of symbiont diversity between host species, but it is tempting to speculate that the spatial structure of *J. wickhami* populations (frequently with small tight clusters of interacting insects on flowering stems) might promote greater horizontal transmission of *Caballeronia* strains and a reduction in the number of common microbial partners at a site (Ravenscraft et al. 2020). What circumstances foster greater horizontal vs. environmental acquisition of the symbiont by *L. zonatus* and how these transmission modes influence the pool of potential symbiont strains is an interesting open question for future study.

### 4.4 Colonization Patterns of *Paraburkholderia* and *Caballeronia* Subclades

Even if bugs passively acquire their symbiont, filtering of ingested microbial mixtures and competition within the M4 region can lead to patterns of *Caballeronia* strain and subclade abundances in the bugs that are not simply a reflection of rank abundances in the local environment (Itoh et al. 2019; Lextrait et al. 2025; Ohbayashi et al. 2022). In a study of competition within bugs of multiple *Burkholderia s.l*. strains, *Caballeronia* strains outcompeted other *Burkholderia* strains that colonized the M4. When introduced to *R. pedestris* nymphs alone, these non‐*Caballeronia* strains caused moderate reductions in fitness (Itoh et al. 2019). In concordance with these findings, we found that *Paraburkholderia* was abundant in soils (~37% of reads) but appeared to be a poor colonizer of wild insects, accounting for less than 1% of *Burkholderia s.l*. sequences from bugs (Figure 2).

Four named subclades of *Caballeronia* have been identified: SBEα, SBEβ, SBE𝛾 and SBE𝛿 (where SBE is an acronym for ‘stinkbug‐associated beneficial and environmental,’ the descriptor assigned to the clade before it was elevated to the genus *Caballeronia*) (Ohbayashi et al. 2022). SBEα and SBEβ were found to confer equivalent benefits to *L. occidentalis* (Ohbayashi et al. 2022) but the SBE𝛾 and SBE𝛿 clades were not evaluated. However, since SBE𝛿 appears to associate largely with insects in the superfamily Coreoidea, prior researchers have speculated that this clade may be particularly highly specialized for symbiosis with this insect group (Kikuchi et al. 2011a; Ohbayashi et al. 2022).

Within the *Caballeronia*, the relative abundances of most subclades were roughly similar in insects versus soils. The SBEα clade was similarly dominant in both insects and soils. The SBE𝛾 clade increased slightly on average in insects relative to soils, while the SBEβ clade decreased slightly. However, the relative abundance of the SBE𝛿 subclade was a major exception: it accounted for 5%–35% of bug symbiont communities across cities, but was nearly absent from soil samples. Further, the common SBE𝛿 lineage, SV001, appears less likely to coexist with other strains in the bug M4 gut section when present (Figures 2 and 3), perhaps suggesting competitive dominance of this strain or subclade once it has colonized the nymph.

Interestingly, in contrast with our findings, the coreid host *Coreus marginatus* most frequently acquired the SBEβ subclade from French soil, and SBEβ strains usually outcompeted SBEα and SBE𝛾 in this insect (SBE𝛿 was not tested; Lextrait et al. 2025). Ohbayashi et al. (2022) also found dominance of the SBEβ subclade and an almost complete absence of the SBEα clade in wild western conifer bug *Leptoglossus occidentalis* , a congener of our study species, in the USA, Europe and Japan, and found that an SBEβ strain outcompeted an SBEα strain when they were introduced to *L. occidentalis* nymphs simultaneously. This suggests the possibility that even closely related insect species may preferentially select for enrichment of different *Caballeronia* subclades within their M4 organs, and/or that the subclades are differentially competitive within different insect species. Fascinatingly, in *Riptortus pedestris* (Alydidae), the subclade that preferentially colonized the insect (SBEα) also confered the greatest reproductive benefit to this host (Lextrait et al. 2025). An exciting area for future research will be to test more broadly whether these subclades are differentially beneficial for different insect species, and whether the competitive dominance of a *Caballeronia* strain or clade within the M4 is correlated with benefits to the bug.

The SBE𝛿 clade is thought to be associated with the insect superfamily Coreoidea, and we found it was the second most abundant subclade in *L. zonatus* (after SBEα). Notably, SBE𝛿 may be even more common in other wild coreid populations or species. In the southeastern United States, we found that this clade accounted for 54% of all *Burkholderia s. l*. reads in 66 *Leptoglossus phyllopus* individuals, 79% of reads in 5 *L. oppositus* individuals, and 94% of reads in 15 *L. fulvicornis* individuals (Figure S6). However, in the lygaeoid (not coreid) bug genus *Jalysus* (Berytidae), SBE𝛿 also dominated wild populations across the United States, accounting for 76% of all *Burkholderia s. l*. reads overall in the insects (Figure S7). Therefore, while different *Leptoglossus* species and different bug clades can preferentially associate with different *Caballeronia* subclades (e.g., Lextrait et al. 2025), further work is needed to understand the taxonomic scale of such preferences: Are particular subclades preferred by different insect families, genera, species or even populations within species?

Variation in the symbiont community hosted by bug taxa will also be driven by varying abundances of *Caballeronia* subclades in local soils. To understand the relative importance of environmental symbiont abundances and preferential associations between particular insect and symbiont clades in determining what symbiont colonizes a bug, it will be necessary for future studies to characterize the composition of *Caballeronia* in soils.

In other systems where the symbiont is recruited from a diverse environmental pool, there is evidence that stochastic effects (Chen et al. 2024) and within‐host competition may influence what strains persist. In the Red Sea Coral, *Stylophora pistillata*, both vertical and horizontal acquisition appear to result in multiple *Symbiodinium* in juvenile corals that are then adaptively selected within the host for persistence of the clade that is superior at a particular water depth (Byler et al. 2013). In the *Vibrio‐*squid system, a clade of ‘dominant’ strains outcompete other strains because they reach the crypts more rapidly, and strains with a type 6 secretion system eliminate strains without it (Visick et al. 2021). The host may also influence strain persistence in the relationship between rhizobium and legumes. Plants may ‘sanction’ nodules containing bacteria that do not fix nitrogen, but they also discriminate among relative symbiont benefits—sanctioning ‘intermediate’ fixers in the presence of better ones, but not when the intermediate fixers are alone (Westhoek et al. 2021). In the *L. zonatus* system, whether bugs are limited by the pool of *Caballeronia* strains present in their environment, and how within‐host filtering and strain interactions affect the benefits the host receives compared to the average quality of available strains, are fascinating open questions.

## 5 Conclusions

A risk of environmental symbiont acquisition for an insect like *L. zonatus* is the possibility of acquiring an inferior symbiont strain (Hunter et al. 2022; Stillson et al. 2025). However, this risk may be balanced by the opportunity to acquire new strains that may be better suited to the local environment. In this context, the distribution of environmental microbes over space and time is likely to have meaningful effects on host fitness. We found that geographic location, much more than time, predictably influences which symbiont lineage is acquired in an environmentally acquired symbiosis. It also appears that certain subclades of *Caballeronia*—particularly the Coreoidea‐associated SBE𝛿 subclade—are enriched in the bugs compared to their presence in the environmental pool for these bacteria, the soil. This could be because strains in this clade are differentially acquired by the insect, or because within the host, these strains are better colonizers of the M4 symbiotic organ, or are better competitors once arriving within the M4.

To further understand symbiotic outcomes in environmentally acquired symbioses, some useful future research on the symbiont side would be to quantify the colonization ability, competitive ability and benefits of different strains and subclades of *Caballeronia* for particular host species and habitats. On the host side, it would be interesting to determine whether hosts actively select certain strains over others, or whether differences in the rank abundance of strains in bugs relative to the local environment are determined by within‐host processes. Finally, it would be interesting to know to what degree hosts ‘stack the deck’ by enriching their local environment with excreted live symbiont cells.

## Author Contributions

A.R. and M.S.H. designed the research; A.R., S.E.K. and J.E.A. performed the research; D.R.H. provided advice on *Leptoglossus* biology, connections to field sites, and facilities during field work; A.R. analysed the data; A.R. and M.S.H. wrote the manuscript; all authors edited the manuscript.

## Funding

This work was supported by an NIH PERT postdoctoral fellowship through the Center for Insect Science to A.R. (grant number K12GM000708); a United States Department of Agriculture (USDA) NIFA grant (2019‐67013‐29407) to M.S.H., A.R. and David Baltrus; a USDA NIFA grant (2023‐67013‐39897) to M.S.H. and A.R.; and an NSF grant (2426306) to M.S.H.

## Conflicts of Interest

The authors declare no conflicts of interest.

## Supporting information


**Figure S1:** Phylogenetic placement of all *Burkholderia s. l*. lineages associated with *Leptoglossus zonatus* and local soils.
**Figure S2:** Percentage of total reads belonging to each *Caballeronia* subclade from bugs and soils in Fresno, Bakersfield and Tucson.
**Figure S3:** Comparison of *Burkholderia s. l*. communities across site types (farms, hedges and nurseries) in (a) insects and (b) soils.
**Figure S4:** Differentially abundant *Caballeronia* lineages among site types.
**Figure S5:** Bray–Curtis dissimilarity between samples at different spatial scales calculated for different site types: (a) hedges and orchards, (b) only orchards and (c) only hedges.
**Figure S6:** Relative abundances of *Burkholderia s. l*. lineages in other *Caballeronia*‐hosting bug species.
**Figure S7:** Relative abundances of *Burkholderia s. l*. in populations of *Jalysus* spp. across the USA.
**Table S1:** Isolate metadata and accession numbers for *Caballeronia* genomes and 16S rRNA sequenced for this study.

## Data Availability

Raw 16s Illumina amplicon sequences are available in the NCBI Sequence Read Archive under Project number PRJNA1144490: https://www.ncbi.nlm.nih.gov/bioproject/PRJNA1144490/. Accession numbers for *Caballeronia* genomes and 16S rRNA sequenced for this study are available in Table S1. All other data and R code are available from the Dryad Digital Repository: https://doi.org/10.5061/dryad.fj6q5743s. Benefits Generated: Benefits from this research accrue from the sharing of our data, results, and data analysis scripts in public databases and repositories as described above. All the research complies with applicable laws on sampling from natural populations and animal experimentation (including the ARRIVE guidelines).

## References

[mec70467-bib-0001] Acevedo, T. S. , G. P. Fricker , J. R. Garcia , et al. 2021. “The Importance of Environmentally‐Acquired Bacterial Symbionts for the Squash Bug ( *Anasa tristis* ), a Significant Agricultural Pest.” bioRxiv. 10.3389/fmicb.2021.719112 PMC8521078 34671328

[mec70467-bib-0002] Baas‐Becking, L. 1934. Geobiologie of Inleiding Tot de Milieukunde (In Dutch). WP Stockum and Zoon.

[mec70467-bib-0003] Bolyen, E. , J. R. Rideout , M. R. Dillon , et al. 2019. “Reproducible, Interactive, Scalable and Extensible Microbiome Data Science Using QIIME 2.” Nature Biotechnology 37: 852–857. 10.1038/s41587-019-0209-9 PMC7015180 31341288

[mec70467-bib-0004] Bull, J. J. 1983. Evolution of Sex Determining Mechanisms. Benjamin/Cummings Publishing Co., Inc.

[mec70467-bib-0005] Byler, K. A. , M. Carmi‐Veal , M. Fine , and T. L. Goulet . 2013. “Multiple Symbiont Acquisition Strategies as an Adaptive Mechanism in the Coral *Stylophora pistillata* .” PLoS One 8: 1–7. 10.1371/journal.pone.0059596 PMC3608662 23555721

[mec70467-bib-0006] Callahan, B. J. , P. J. McMurdie , M. J. Rosen , A. W. Han , A. J. A. Johnson , and S. P. Holmes . 2016. “DADA2: High‐Resolution Sample Inference From Illumina Amplicon Data.” Nature Methods 13, no. 7: 581–583. 10.1038/nmeth.3869. 27214047 PMC4927377

[mec70467-bib-0007] Chen, I. M. A. , K. Chu , K. Palaniappan , et al. 2023. “The IMG/M Data Management and Analysis System v.7: Content Updates and New Features.” Nucleic Acids Research 51: D723–D732. 36382399 10.1093/nar/gkac976 PMC9825475

[mec70467-bib-0008] Chen, J. Z. , Z. Kwong , N. M. Gerardo , and N. M. Vega . 2024. “Ecological Drift During Colonization Drives Within‐Host and Between‐Host Heterogeneity in an Animal‐Associated Symbiont.” PLoS Biology 22: e3002304. 38662791 10.1371/journal.pbio.3002304 PMC11075893

[mec70467-bib-0009] Doremus, M. R. , S. E. Kelly , and M. S. Hunter . 2019. “Exposure to Opposing Temperature Extremes Causes Comparable Effects on *Cardinium* Density but Contrasting Effects on *Cardinium*‐Induced Cytoplasmic Incompatibility.” PLoS Pathogens 15: e1008022. 31425566 10.1371/journal.ppat.1008022 PMC6715252

[mec70467-bib-0010] Engel, P. , and N. A. Moran . 2013. “The Gut Microbiota of Insects ‐ Diversity in Structure and Function.” FEMS Microbiology Reviews 37: 699–735. 23692388 10.1111/1574-6976.12025

[mec70467-bib-0011] Fronk, D. C. , and J. L. Sachs . 2022. “Symbiotic Organs: The Nexus of Host‐Microbe Evolution.” Trends in Ecology & Evolution 37: 599–610. 35393155 10.1016/j.tree.2022.02.014

[mec70467-bib-0012] Garcia, J. R. , A. M. Laughton , Z. Malik , et al. 2014. “Partner Associations Across Sympatric Broad‐Headed Bug Species and Their Environmentally Acquired Bacterial Symbionts.” Molecular Ecology 23: 1333–1347. 24384031 10.1111/mec.12655

[mec70467-bib-0013] Hamady, M. , J. J. Walker , J. K. Harris , N. J. Gold , and R. Knight . 2008. “Error‐Correcting Barcoded Primers for Pyrosequencing Hundreds of Samples in Multiplex.” Nature Methods 5, no. 3: 235–237. 10.1038/NMETH.1184. 18264105 PMC3439997

[mec70467-bib-0014] Hassler, H. B. , B. Probert , C. Moore , et al. 2022. “Phylogenies of the 16S rRNA Gene and Its Hypervariable Regions Lack Concordance With Core Genome Phylogenies.” Microbiome 10: 104. 35799218 10.1186/s40168-022-01295-y PMC9264627

[mec70467-bib-0015] Hehemann, J. H. , G. Correc , T. Barbeyron , W. Helbert , M. Czjzek , and G. Michel . 2010. “Transfer of Carbohydrate‐Active Enzymes From Marine Bacteria to Japanese Gut Microbiota.” Nature 464: 908–912. 20376150 10.1038/nature08937

[mec70467-bib-0016] Hoegh‐Guldberg, O. 1999. “Climate Change, Coral Bleaching and the Future of the World's Coral Reefs.” Marine and Freshwater Research 50: 839–866.

[mec70467-bib-0017] Hunter, M. S. , E. F. Umanzor , S. E. Kelly , S. M. Whitaker , and A. Ravenscraft . 2022. “Development of Common Leaf‐Footed Bug Pests Depends on the Presence and Identity of Their Environmentally Acquired Symbionts.” Applied and Environmental Microbiology 88: e01778. 34986009 10.1128/aem.01778-21 PMC8904059

[mec70467-bib-0018] Ingels, C. , and D. Haviland . 2014. “Leaffooted Bug.” [Online]. Accessed UC ANR Publication 74168. http://ipm.ucanr.edu/PDF/PESTNOTES/pnleaffootedbug.pdf.

[mec70467-bib-0019] Itoh, H. , S. Jang , K. Takeshita , et al. 2019. “Host–Symbiont Specificity Determined by Microbe–Microbe Competition in an Insect Gut.” Proceedings of the National Academy of Sciences of the United States of America 116: 22673–22682. 31636183 10.1073/pnas.1912397116 PMC6842582

[mec70467-bib-0020] Itoh, H. , R. Navarro , K. Takeshita , et al. 2014. “Bacterial Population Succession and Adaptation Affected by Insecticide Application and Soil Spraying History.” Frontiers in Microbiology 5: 457. 25221549 10.3389/fmicb.2014.00457 PMC4148734

[mec70467-bib-0021] Itoh, H. , K. Tago , M. Hayatsu , and Y. Kikuchi . 2018. “Detoxifying Symbiosis: Microbe‐Mediated Detoxification of Phytotoxins and Pesticides in Insects.” Natural Product Reports 35: 434–454. 29644346 10.1039/c7np00051k

[mec70467-bib-0022] Jang, S. , and Y. Kikuchi . 2020. “Impact of the Insect Gut Microbiota on Ecology, Evolution, and Industry.” Current Opinion in Insect Science 41: 33–39. 32634703 10.1016/j.cois.2020.06.004

[mec70467-bib-0023] Joyce, A. L. , B. S. Higbee , D. R. Haviland , and H. Brailovsky . 2017. “Genetic Variability of Two Leaffooted Bugs, *Leptoglossus clypealis* and *Leptoglossus zonatus* (Hemiptera: Coreidae) in the Central Valley of California.” Journal of Economic Entomology 110: 2576–2589. 29045641 10.1093/jee/tox222

[mec70467-bib-0024] Katoh, K. , and D. M. Standley . 2013. “MAFFT Multiple Sequence Alignment Software Version 7: Improvements in Performance and Usability.” Molecular Biology and Evolution 30, no. 4: 772–780. 23329690 10.1093/molbev/mst010 PMC3603318

[mec70467-bib-0025] Kikuchi, Y. , M. Hayatsu , T. Hosokawa , A. Nagayama , K. Tago , and T. Fukatsu . 2012. “Symbiont‐Mediated Insecticide Resistance.” Proceedings of the National Academy of Sciences of the United States of America 109: 8618–8622. 22529384 10.1073/pnas.1200231109 PMC3365206

[mec70467-bib-0026] Kikuchi, Y. , T. Hosokawa , and T. Fukatsu . 2007. “Insect‐Microbe Mutualism Without Vertical Transmission: A Stinkbug Acquires a Beneficial Gut Symbiont From the Environment Every Generation.” Applied and Environmental Microbiology 73: 4308–4316. 17483286 10.1128/AEM.00067-07 PMC1932760

[mec70467-bib-0027] Kikuchi, Y. , T. Hosokawa , and T. Fukatsu . 2011a. “An Ancient but Promiscuous Host‐Symbiont Association Between *Burkholderia* Gut Symbionts and Their Heteropteran Hosts.” ISME Journal 5: 446–460. 20882057 10.1038/ismej.2010.150 PMC3105724

[mec70467-bib-0028] Kikuchi, Y. , T. Hosokawa , and T. Fukatsu . 2011b. “Specific Developmental Window for Establishment of an Insect‐Microbe Gut Symbiosis.” Applied and Environmental Microbiology 77, no. 12: 4075–4081. 21531836 10.1128/AEM.00358-11 PMC3131632

[mec70467-bib-0029] Kikuchi, Y. , X. Y. X. Meng , and T. Fukatsu . 2005. “Gut Symbiotic Bacteria of the Genus *Burkholderia* in the Broad‐Headed Bugs *Riptortus clavatus* and *Leptocorisa chinensis* (Heteroptera: Alydidae).” Applied and Environmental Microbiology 71, no. 7: 4035–4043. 16000818 10.1128/AEM.71.7.4035-4043.2005 PMC1169019

[mec70467-bib-0030] Kinosita, Y. , Y. Kikuchi , N. Mikami , D. Nakane , and T. Nishizaka . 2018. “Unforeseen Swimming and Gliding Mode of an Insect Gut Symbiont, Burkholderia sp. RPE64, With Wrapping of the Flagella Around Its Cell Body.” ISME Journal 12: 838–848. 29269839 10.1038/s41396-017-0010-z PMC5864224

[mec70467-bib-0031] Kuechler, S. M. , Y. Matsyuura , K. Dettner , and Y. Kikuchi . 2016. “Phylogenetically Diverse Burkholderia Associated With Midgut Crypts of Spurge Bugs, Dicranocephalus spp. (Heteroptera: Stenocephalidae).” Microbes and Environments 31: 145–153. 27265344 10.1264/jsme2.ME16042 PMC4912149

[mec70467-bib-0032] Lextrait, G. , S. Joardar , R. Cossard , Y. Kikuchi , T. Ohbayashi , and P. Mergaert . 2025. “Strict Gut Symbiont Specificity in Coreoidea Insects Governed by Interspecies Competition Within *Caballeronia* Strains.” ISME Journal 19, no. 1: wraf240. 41230826 10.1093/ismejo/wraf240 PMC12632191

[mec70467-bib-0033] Martin, M. 2011. “Cutadapt Removes Adapter Sequences From High‐Throughput Sequencing Reads.” EMBnet.Journal 17, no. 1: 10. 10.14806/ej.17.1.200.

[mec70467-bib-0034] Mason, C. J. , J. J. Couture , and K. F. Raffa . 2014. “Plant‐Associated Bacteria Degrade Defense Chemicals and Reduce Their Adverse Effects on an Insect Defoliator.” Oecologia 175: 901–910. 24798201 10.1007/s00442-014-2950-6

[mec70467-bib-0035] McCutcheon, J. P. , and N. A. Moran . 2012. “Extreme Genome Reduction in Symbiotic Bacteria.” Nature Reviews Microbiology 10: 13–26. 10.1038/nrmicro2670 22064560

[mec70467-bib-0036] Mcfall‐Ngai, M. , M. G. Hadfield , T. C. G. Bosch , et al. 2013. “Animals in a Bacterial World, a New Imperative for the Life Sciences.” Proceedings of the National Academy of Sciences of the United States of America 110: 3229–3236. 23391737 10.1073/pnas.1218525110 PMC3587249

[mec70467-bib-0037] McMurdie, P. J. , and S. Holmes . 2013. “Phyloseq: An R Package for Reproducible Interactive Analysis and Graphics of Microbiome Census Data.” PLoS One 8, no. 4: e61217. 23630581 10.1371/journal.pone.0061217 PMC3632530

[mec70467-bib-0038] Mirarab, S. , N. Nguyen , and T. Warnow . 2012. “SEPP: SATe‐Enabled Phylogenetic Placement.” 17th Pacific Symposium on Biocomputing (PSB), Jan 03–07 2012, Kohala Coast, HI, 247–258. 10.1142/9789814366496_0024 22174280

[mec70467-bib-0039] Moran, N. A. , P. Tran , and N. M. Gerardo . 2005. “Symbiosis and Insect Diversification: An Ancient Symbiont of Sap‐Feeding Insects From the Bacterial Phylum Bacteroidetes.” Applied and Environmental Microbiology 71: 8802–8810. 16332876 10.1128/AEM.71.12.8802-8810.2005 PMC1317441

[mec70467-bib-0040] Ohbayashi, T. , R. Cossard , G. Lextrait , et al. 2022. “Intercontinental Diversity of *Caballeronia* Gut Symbionts in the Conifer Pest Bug *Leptoglossus occidentalis* .” Microbes and Environments 37: n/a. 10.1264/jsme2.ME22042 PMC9530724 35965097

[mec70467-bib-0041] Ohbayashi, T. , R. Futahashi , M. Terashima , et al. 2019. “Comparative Cytology, Physiology and Transcriptomics of *Burkholderia insecticola* in Symbiosis With the Bean Bug *Riptortus pedestris* and in Culture.” ISME Journal 13: 1469–1483. 30742016 10.1038/s41396-019-0361-8 PMC6776119

[mec70467-bib-0042] Ohbayashi, T. , K. Takeshita , W. Kitagawa , et al. 2015. “Insect's Intestinal Organ for Symbiont Sorting.” Proceedings of the National Academy of Sciences of the United States of America 112: E5179‐E5188. 26324935 10.1073/pnas.1511454112 PMC4577176

[mec70467-bib-0043] O'Neill, S. L. , A. A. Hoffmann , and J. H. Werren . 1997. Influential Passengers. Oxford University Press.

[mec70467-bib-0065] Parajuli, B. S. , J. Teodosio , and A. Ravenscraft . 2026. “Leaffooted Bugs Enrich Local Soil With Their Horizontally Acquired Symbiont.” Frontiers in Microbiology 17: 1737071. 42338892 10.3389/fmicb.2026.1737071 PMC13284837

[mec70467-bib-0044] Quast, C. , E. Pruesse , P. Yilmaz , et al. 2013. “The SILVA Ribosomal RNA Gene Database Project: Improved Data Processing and Web‐Based Tools.” Nucleic Acids Research 41, no. D1: 590–596. 10.1093/nar/gks1219 PMC3531112 23193283

[mec70467-bib-0045] Ravenscraft, A. , M. Berry , T. Hammer , K. Peay , and C. Boggs . 2019. “Structure and Function of the Bacterial and Fungal Gut Microbiota of Neotropical Butterflies.” Ecological Monographs 89, no. 2: e01346.

[mec70467-bib-0064] Ravenscraft, A. , and K. L. Coon . 2025. “Transient Microbes in Insects: Fleeting but Functional.” Annual Review of Entomology 71. 10.1146/annurev-ento-121423-013446 41082399

[mec70467-bib-0046] Ravenscraft, A. , M. W. Thairu , A. K. Hansen , and M. S. Hunter . 2020. “Continent‐Scale Sampling Reveals Fine‐Scale Turnover in a Beneficial Bug Symbiont.” Frontiers in Microbiology 11: 1276. 32636818 10.3389/fmicb.2020.01276 PMC7316890

[mec70467-bib-0063] Rohland, N. , and D. Reich . 2012. “Cost‐Effective, High‐Throughput DNA Sequencing Libraries for Multiplexed Target Capture.” Genome Research 22: 939–946. 10.1101/gr.128124.111.22. 22267522 PMC3337438

[mec70467-bib-0047] Russell, J. A. , and N. A. Moran . 2006. “Costs and Benefits of Symbiont Infection in Aphids: Variation Among Symbionts and Across Temperatures.” Proceedings. Biological Sciences / the Royal Society 273: 603–610. 10.1098/rspb.2005.3348 PMC1560055 16537132

[mec70467-bib-0048] Russell, S. L. 2019. “Transmission Mode Is Associated With Environment Type and Taxa Across Bacteria‐Eukaryote Symbioses: A Systematic Review and meta‐Analysis.” FEMS Microbiology Letters 366: fnz013. 30649338 10.1093/femsle/fnz013

[mec70467-bib-0049] Stamatakis, A. 2014. “RAxML Version 8: A Tool for Phylogenetic Analysis and Post‐Analysis of Large Phylogenies.” Bioinformatics 30, no. 9: 1312–1313. 24451623 10.1093/bioinformatics/btu033 PMC3998144

[mec70467-bib-0050] Stillson, P. T. , K. Martinez , J. Adamson , A. Tehrani , and A. Ravenscraft . 2025. “Temperature Influences Outcomes of an Environmentally Acquired Symbiosis.” ISME Journal 19, no. 1: wraf056. 40116466 10.1093/ismejo/wraf056 PMC11995993

[mec70467-bib-0051] Stoy, K. S. , J. Chavez , V. De Las Casas , et al. 2023. “Evaluating Coevolution in a Horizontally Transmitted Mutualism.” Evolution 77: 166–185. 36622711 10.1093/evolut/qpac009

[mec70467-bib-0052] Tago, K. , H. Itoh , Y. Kikuchi , et al. 2014. “A Fine‐Scale Phylogenetic Analysis of Free‐Living Burkholderia Species in Sugarcane Field Soil.” Microbes and Environments 29, no. 4: 434–437. 25410730 10.1264/jsme2.ME14122 PMC4262370

[mec70467-bib-0053] Tago, K. , T. Okubo , H. Itoh , et al. 2015. “Insecticide‐Degrading Burkholderia Symbionts of the Stinkbug Naturally Occupy Various Environments of Sugarcane Fields in a Southeast Island of Japan.” Microbes and Environments 30: 29–36. 25736865 10.1264/jsme2.ME14124 PMC4356461

[mec70467-bib-0054] Takeshita, K. , Y. Matsuura , H. Itoh , et al. 2015. “Burkholderia of Plant‐Beneficial Group Are Symbiotically Associated With Bordered Plant Bugs (Heteroptera: Pyrrhocoroidea: Largidae).” Microbes and Environments 30: 321–329. 26657305 10.1264/jsme2.ME15153 PMC4676555

[mec70467-bib-0055] Takeshita, K. , T. F. Shibata , N. Nikoh , et al. 2014. “Whole‐Genome Sequence of Burkholderia sp. Strain RPE67, a Bacterial Gut Symbiont of the Bean Bug *Riptortus pedestris* .” Genome Announcements 2, no. 3: e00556‐14. 24948758 10.1128/genomeA.00556-14 PMC4064023

[mec70467-bib-0066] Umanzor, E. F. , S. E. Kelly , A. Ravenscraft , Y. Matsuura , and M. S. Hunter . 2025. “The Facultative Intracellular Symbiont Lariskella is Neutral for Lifetime Fitness and Spreads Through Cytoplasmic Incompatibility in the Leaffooted Bug, *Leptoglossus zonatus* .” Frontiers in Microbiology 16. 10.3389/fmicb.2025.1595917. PMC12288687 40708921

[mec70467-bib-0056] Villa, S. M. , J. Z. Chen , Z. Kwong , A. Acosta , N. M. Vega , and N. M. Gerardo . 2023. “Specialized Acquisition Behaviors Maintain Reliable Environmental Transmission in an Insect‐Microbial Mutualism.” Current Biology 33: 2830. 37385254 10.1016/j.cub.2023.05.062

[mec70467-bib-0057] Visick, K. L. , E. V. Stabb , and E. G. Ruby . 2021. “A Lasting Symbiosis: How *Vibrio fischeri* Finds a Squid Partner and Persists Within Its Natural Host.” Nature Reviews Microbiology 19: 654–665. 34089008 10.1038/s41579-021-00557-0 PMC8529645

[mec70467-bib-0058] Wang, Q. , G. M. Garrity , J. M. Tiedje , and J. R. Cole . 2007. “Naïve Bayesian Classifier for Rapid Assignment of rRNA Sequences Into the New Bacterial Taxonomy.” Applied and Environmental Microbiology 73, no. 16: 5261–5267. 17586664 10.1128/AEM.00062-07 PMC1950982

[mec70467-bib-0059] Wernegreen, J. J. 2012. “Mutualism Meltdown in Insects: Bacteria Constrain Thermal Adaptation.” Current Opinion in Microbiology 15: 255–262. 22381679 10.1016/j.mib.2012.02.001 PMC3590105

[mec70467-bib-0060] Westhoek, A. , L. J. Clark , M. Culbert , et al. 2021. “Conditional Sanctioning in a *Legume‐Rhizobium* Mutualism.” Proceedings of the National Academy of Sciences of the United States of America 118, no. 19: e2025760118. 33941672 10.1073/pnas.2025760118 PMC8126861

[mec70467-bib-0061] Xu, Y. , E. A. Buss , and D. G. Boucias . 2016. “Culturing and Characterization of Gut Symbiont Burkholderia spp. From the Southern Chinch Bug, *Blissus insularis* (Hemiptera: Blissidae).” Applied and Environmental Microbiology 82: 3319–3330. 27016568 10.1128/AEM.00367-16 PMC4959241

[mec70467-bib-0062] Yang, P. , and J. D. Van Elsas . 2018. “Mechanisms and Ecological Implications of the Movement of Bacteria in Soil.” Applied Soil Ecology 129: 112–120.

