# Supplementary material for: Spatial and Temporal Structure of Environmentally‐Acquired Caballeronia Symbionts of a Leaffooted Bug
Source: Mol Ecol. 2026 Jul 27;35(14):e70467. doi: 10.1111/mec.70467 (PMC13403611; doi:10.1111/mec.70467)
Supplement: Supplementary file 1 — Figure S1: Phylogenetic placement of all Burkholderia s. l. lineages associated with Leptoglossus zonatus and local soils. Figure S2: Percentage of total reads belonging to each Caballeronia subclade from bugs and soils in Fresno, Bakersfield and Tucson. Figure S3: Comparison of Burkholderia s. l. communities across site types (farms, hedges and nurseries) in (a) insects and (b) soils. Figure S4: Differentially abundant Caballeronia lineages among site types. Figure S5: Bray–Curtis dissimilarity between samples at different spatial scales calculated for different site types: (a) hedges and orchards, (b) only orchards and (c) only hedges. Figure S6: Relative abundances of Burkholderia s. l. lineages in other Caballeronia‐hosting bug species. Figure S7: Relative abundances of Burkholderia s. l. in populations of Jalysus spp. across the USA. Table S1: Isolate metadata and accession numbers for Caballeronia genomes and 16S rRNA sequenced for this study. [file MEC-35-e70467-s001.docx]

**Supplemental Information for:**

**Spatial and temporal structure of environmentally-acquired**

***Caballeronia* symbionts of a leaffooted bug**

Alison Ravenscraft, Suzanne E. Kelly, David R. Haviland, Johnathan E. Adamson, Martha S. Hunter

**Table of Contents:**

| **Fig S1. Phylogenetic placement of all *Burkholderia s. l.* lineages** | Page 2 |
| --- | --- |
| **Fig S2. *Caballeronia* subclade proportions across sites** | Page 3 |
| **Fig S3. Comparison of *Burkholderia s. l.* communities across site types** | Page 4 |
| **Fig S4.** **Differentially abundant *Caballeronia* lineages among site types** | Page 5 |
| **Fig S5. Bray-Curtis dissimilarity between samples different spatial**  **scales calculated for different site types** | Page 6 |
| **Fig S6. *Burkholderia s. l.* lineages in other bug species** | Page 7 |
| **Fig S7. *Burkholderia s. l.* lineages in stilt bugs (*Jalysus* spp.)** | Page 8 |
| **Table S1. Isolate metadata and accession numbers of sequences**  **generated for this study** | Page 9-12 |

**Figure S1.** Phylogenetic placement of all *Burkholderia s. l.* lineages associated with *Leptoglossus zonatus* and local soils. This figure is identical to Figure 1 (main text), except that all lineages (including those that did not account for at least 1% of the reads in at least 2 samples) and all reference sequences are shown. Accession numbers for the reference sequences are available in the phylogeny metadata on Dryad.


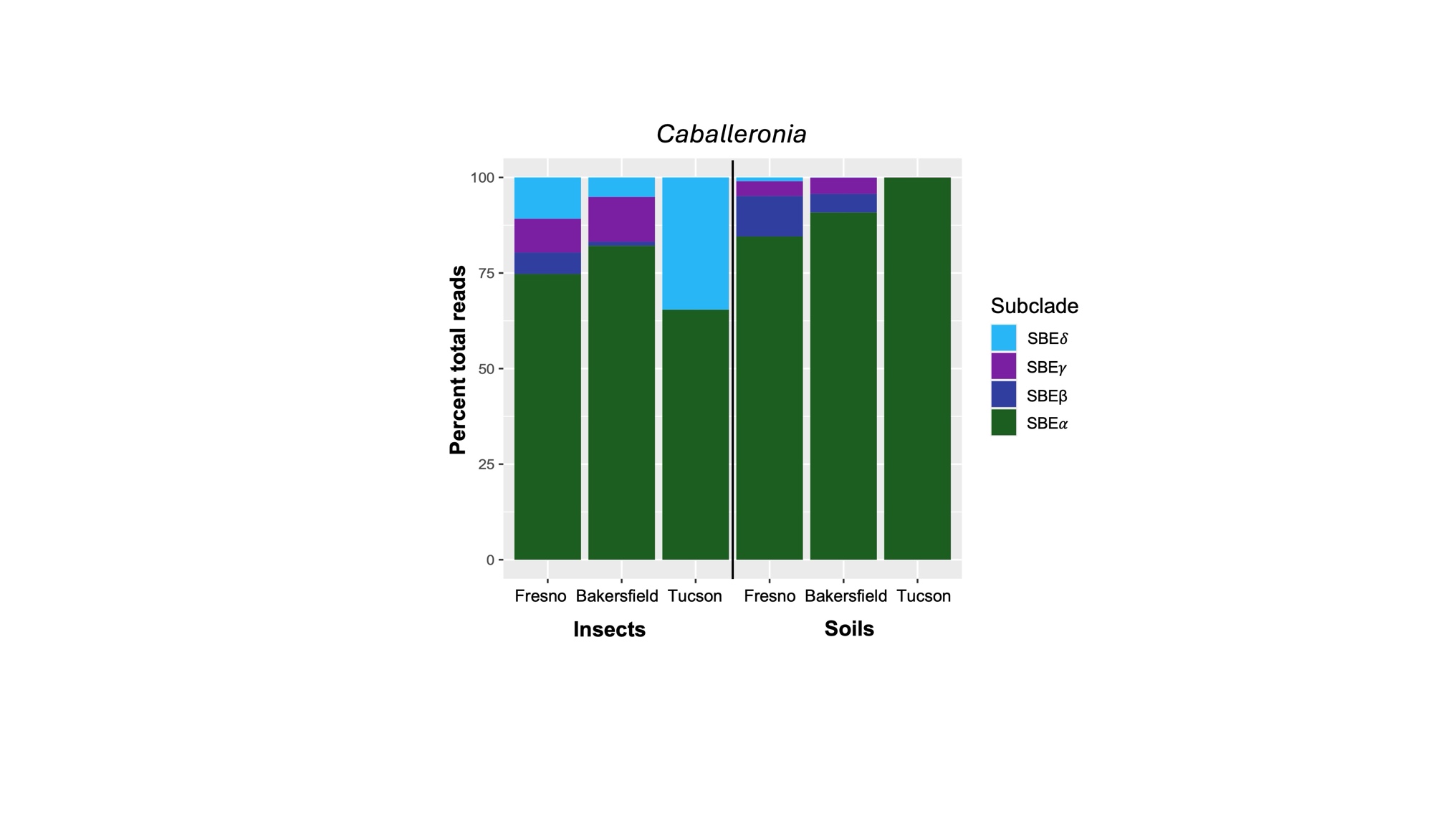


**Figure S2.** Percentage of total reads belonging to each *Caballeronia* subclade from bugs and soils in Fresno, Bakersfield and Tucson. Bug percentages from Tucson include insects sampled from one pomegranate orchard over the course of two years. An equivalent chart for all *Burkholderia s. l.* is available in Figure 2 (main text).

**
Figure S3.** Comparison of *Burkholderia s. l.* communities across site types (farms, hedges and nurseries) in (a) insects and (b) soils. Farms samples were from organic pomegranate orchards; hedge samples were from wild unmaintained boarders of pomegranate next to roads; nursery samples were from potted pomegranate trees. Figure features are the same as in Fig 3 in the main text.

**Figure S4.** Differentially abundant *Caballeronia* lineages among site types. Each point represents abundance of a lineage in a single sample, with point color indicating the collection location: Tucson, AZ sites are in shades of green, Fresno, CA sites are in shades of orange, and Bakersfield, CA sites are in shares of purple. There were three differentially abundant lineages in insects (a) and five in soils (b), as determined by the DESeq2 test for differential abundance adapted for microbiome data (McMurdie P.J. & Holmes S., 2014, *PLoS computational biology*, *10*(4), e1003531). However, site type and location tended to be confounded.

**Figure S5.** Bray-Curtis dissimilarity between samples at different spatial scales calculated for different site types: (a) hedges and orchards, (b) only orchards, and (c) only hedges. The results were qualitatively identical to those in the main text (Fig 4) when samples from nurseries were omitted (a). For just orchards and just hedges, we did not have enough statistical power to detect differences between soil communities at different scales, but the trends agreed with those in the main text.

Every point represents the Bray-Curtis dissimilarity in *Caballeronia* lineage composition between a pair of bugs (left panels), a bug and a soil sample (middle panels), or a pair of soil samples (right panels). Pairwise dissimilarities are grouped according to whether the samples came from the same tree, different trees in the same site, different sites in the same city, or different cities. Box plots depict medians and interquartile ranges of the data. Whiskers are placed at 1.5 times the interquartile range or, if all data fall within this range, they are placed at most extreme value measured. Letters above the box plots indicate statistically significant differences at p<0.05. The Bray-Curtis metric ranges from a score of 0, which indicates that two samples share identical lineages at identical relative abundances, to a score of 1, indicating that two samples don’t share any lineage in common.

**Figure S6.** Relative abundances of *Burkholderia* *s. l.* lineages in other *Caballeronia*-hosting bug species. Each bar represents an individual insect and all samples were rarefied to 2179 reads. Samples are sorted according to the abundances of the lineages, with the most abundant sorted first, followed by the second most abundant, etc. Color scale and lineages are the same as those appearing in Figs 3 and 6. Samples were prepared as described in the main text for *L. zonatus* and were sequenced on the same Illumina run. L. br = *Leptoglossus brevirostris*. L. op = *Leptoglossus oppositus*. *Leptoglossus brevirostris* was collected in Arizona in 2018. All other individuals were collected in South Carolina in July 2018.

**Figure S7.** Relative abundances of *Burkholderia* *s. l.* in populations of *Jalysus* spp. across the USA. Sequence variants were placed on the reference phylogeny (described in main text) and grouped into lineages at a cophenetic distance of 0.1. Each bar represents an insect. Samples were rarefied to 2179 reads. Plots are titled with the collection site plus the genus of the host plant or a description of the habitat. Samples are sorted according to the abundances of the lineages, with the most abundant sorted first, followed by the second most abundant, etc. This figure is adapted from Fig. 3 of Ravenscraft *et al.,* 2020; the details of insect collection, preparation and sequencing are reported there. The primer set only partially overlaps the amplicon region in the main study, so lineages here cannot be conclusively matched to lineages in other figures. However, phylogenetic placement suggests that:

jal18 is closely related to sv019

jal16 is identical to sv002

jal17 is closely related to sv024

jal53 is identical to sv038

jal43 is closely related to sv106

jal22 is closely related to sv012

jal173 is identical to sv004

jal1 is identical to sv001

jal35 is closely related to sv436 and sv153

jal24 is closely related to sv037

jal14 has no close relatives in *Leptoglossus*

jal132 is identical to sv035

Ravenscraft, A., Thairu, M. W., Hansen, A. K. & Hunter, M. S. (2020) Continent-scale sampling reveals fine-scale turnover in a beneficial bug symbiont. *Frontiers in Microbiology,* 11**,** 1276

**Table S1.** Isolate metadata and accession numbers for *Caballeronia* genomes and 16S rRNA sequenced for this study

| **Isolate** | **Description** | **Host insect** | **Accession** | **Clade** | **Most closely related Illumina lineage** | **sequence type** | **host plant** | **state** | **region** | **patch** | **bugindiv** | **Genome sequencing and assembly** |
| --- | --- | --- | --- | --- | --- | --- | --- | --- | --- | --- | --- | --- |
| Lep1A1 | Caballeronia grimmiae Lep1A1 | Leptglossus zonatus | GCA_022879615 | SBEa | sv012 | genome | tomato/ pomegranate | AZ/CA | AZ lab colony started from CA bugs | Lep1 | see Stillson et al 2022 |  |
| Lep1P3 | Caballeronia sp Lep1P3 | Leptglossus zonatus | GCA_022879595 | SBEa | sv333 | genome | tomato/ pomegranate | AZ/CA | AZ lab colony started from CA bugs | Lep1 | see Stillson et al 2022 |  |
| LP003 | LP003 | Leptoglossus phyllopus | PQ197222 | SBEa | sv002 | 16S | Solanum ptycanthum | TX | DFW |  | LP003 | n/a |
| LP004c9 | LP004c9 | Leptoglossus phyllopus | PQ197223 | SBEd | sv001 | 16S | Solanum ptycanthum | TX | DFW |  | LP004 | n/a |
| LP006 | Caballeronia sp LP006 | Leptoglossus phyllopus | GCA_031450995 | SBEd | sv001 | genome | Solanum ptycanthum | TX | DFW |  | LP006 | Plasmidsaurus^2^ |
| LZ001 | Caballeronia sp LZ001 | Leptglossus zonatus | GCA_031450875 | SBEd | sv001 | genome | pomegranate | AZ | tucson | MV | MVd1 | SNPsaurus^1^ |
| LZ002 | Caballeronia sp LZ002 | Leptglossus zonatus | GCA_031451575 | SBEd | sv001 | genome | pomegranate | AZ | tucson | MV | MVc7 | SNPsaurus^1^ |
| LZ003 | Caballeronia sp LZ003 | Leptglossus zonatus | GCA_031451465 | SBEd | sv001 | genome | pomegranate | AZ | tucson | MV | MVc7 | SNPsaurus^1^ |
| LZ004 | LZ004 | (soil near L. zonatus) | PQ197225 | Cupriavidus | none | 16S | soil under pomegranate | AZ | tucson | MG | (soil MGd) | n/a |
| LZ006 | LZ006 | Leptglossus zonatus | PQ197226 | SBEd | sv001 | 16S | pomegranate | AZ | tucson | MV | MVd1 | n/a |
| LZ008 | Caballeronia sp LZ008 | Leptglossus zonatus | GCA_031451435 | SBEa | sv002 | genome | pomegranate | AZ | tucson | MG | MGf7 | SNPsaurus^1^ |
| LZ009 | LZ009 | Leptglossus zonatus | PQ197227 | SBEd | sv001 | 16S | pomegranate | AZ | tucson | MV | MVc7 | n/a |
| LZ010 | LZ010 | (soil near L. zonatus) | PQ197228 | Cupriavidus | none | 16S | soil under pomegranate | AZ | tucson | UA | (soil UAc) | n/a |
| LZ012 | LZ012 | (soil near L. zonatus) | PQ197229 | Cupriavidus | none | 16S | soil under pomegranate | AZ | tucson | MG | (soil MGd) | n/a |
| LZ013 | LZ013 | Leptglossus zonatus | PQ197230 | SBEa | sv004 | 16S | pomegranate | AZ | tucson | MG | MGe7 | n/a |
| LZ014 | LZ014 | Leptglossus zonatus | PQ197231 | SBEd | sv001 | 16S | pomegranate | AZ | tucson | UA | UAa8 | n/a |
| LZ015 | LZ015 | Leptglossus zonatus | PQ197232 | SBEa | sv002 | 16S | pomegranate | AZ | tucson | MG | MGf7 | n/a |
| LZ016 | Caballeronia sp LZ016 | Leptglossus zonatus | GCA_031450805 | SBEa | sv010 | genome | pomegranate | AZ | tucson | MG | MGf7 | Plasmidsaurus^2^ |
| LZ017 | LZ017 | Leptglossus zonatus | PQ197233 | SBEd | sv001 | 16S | pomegranate | AZ | tucson | UA | UAb8 | n/a |
| LZ018 | LZ018 | Leptglossus zonatus | PQ197234 | SBEa | sv004 | 16S | pomegranate | AZ | tucson | UA | UAc9 | n/a |
| LZ019 | Caballeronia sp LZ019 | Leptglossus zonatus | GCA_031450825 | SBEa | sv010 | genome | pomegranate | AZ | tucson | MG | MGe7 | Plasmidsaurus^2^ |
| LZ020 | LZ020 | Leptglossus zonatus | PQ197235 | SBEa | sv004 | 16S | pomegranate | AZ | tucson | UA | UAc9 | n/a |
| LZ022 | LZ022 | Leptglossus zonatus | PQ197236 | SBEa | sv002 | 16S | pomegranate | AZ | tucson | MG | MGf7 | n/a |
| LZ024 | Caballeronia sp LZ024 | Leptglossus zonatus | GCA_031451455 | SBEg | sv020 | genome | pomegranate | CA | fresno | RE | REa1 | SNPsaurus^1^ |
| LZ025 | Caballeronia sp LZ025 | Leptglossus zonatus | GCA_031451475 | SBEa | sv012 | genome | pomegranate | CA | bakersfield | WD | WDb9 | SNPsaurus^1^ |
| LZ026 | LZ026 | Leptglossus zonatus | PQ197237 | SBEd | sv001 | 16S | pomegranate | CA | bakersfield | WF | WFc7 | n/a |
| LZ027 | LZ027 | Leptglossus zonatus | PQ197238 | SBEa | sv010 | 16S | pomegranate | AZ | tucson | MG | MGf7 | n/a |
| LZ028 | Caballeronia sp LZ028 | Leptglossus zonatus | GCF_031451415 | SBEa | sv002 | genome | pomegranate | CA | bakersfield | WF | WFb8 | SNPsaurus^1^ |
| LZ029 | Caballeronia sp LZ029 | Leptglossus zonatus | GCA_031451275 | SBEa | sv458 | genome | pomegranate | CA | bakersfield | WR | WRb1 | SNPsaurus^1^ |
| LZ030 | LZ030 | Leptglossus zonatus | PQ197239 | SBEd | sv001 | 16S | pomegranate | CA | bakersfield | WF | WFc7 | n/a |
| LZ031 | Caballeronia sp LZ031 | Leptglossus zonatus | GCA_031450775 | SBEg | sv020 | genome | pomegranate | CA | fresno | RE | REa1 | Plasmidsaurus^2^ |
| LZ032 | Caballeronia sp LZ032 | Leptglossus zonatus | GCA_031451315 | SBEa | sv004 | genome | pomegranate | CA | fresno | RE | REc1 | SNPsaurus^1^ |
| LZ033 | Caballeronia sp LZ033 | Leptglossus zonatus | GCA_031451265 | SBEa | sv004 | genome | pomegranate | CA | fresno | KA | KAb1 | SNPsaurus^1^ |
| LZ034LL | Caballeronia LZ034LL | Leptglossus zonatus | GCA_031451215 | SBEa | sv004 | genome | pomegranate | AZ | tucson | UA | UAc9 | SNPsaurus^1^ |
| LZ035 | Caballeronia LZ035 | Leptglossus zonatus | GCA_031451175 | SBEa | sv016/sv241 | genome | pomegranate | CA | fresno | ET | ETb1 | SNPsaurus^1^ |
| LZ037 | LZ037 | Leptglossus zonatus | PQ197240 | SBEa | sv012 | 16S | pomegranate | CA | bakersfield | WD | WDb9 | n/a |
| LZ038 | LZ038 | Leptglossus zonatus | PQ197241 | SBEa | sv002 | 16S | pomegranate | AZ | tucson | MV | MVc6 | n/a |
| LZ041 | LZ041 | Leptglossus zonatus | PQ197242 | SBEa | sv016 | 16S | pomegranate | CA | fresno | RE | REd1 | n/a |
| LZ042 | LZ042 | Leptglossus zonatus | PQ197243 | SBEa | sv016 | 16S | pomegranate | CA | fresno | ET | ETb1 | n/a |
| LZ043 | Caballeronia sp LZ043 | Leptglossus zonatus | GCA_031451075 | SBEa | sv004 | genome | pomegranate | CA | fresno | ET | ETa1 | SNPsaurus^1^ |
| LZ046 | LZ046 | Leptglossus zonatus | PQ197244 | SBEa | sv016 | 16S | pomegranate | CA | bakersfield | WD | WDa7 | n/a |
| LZ049B | LZ049B | Leptglossus zonatus | PQ197245 | SBEd | sv001 | 16S | pomegranate | AZ | tucson | UA | UAa8 | n/a |
| LZ049L | LZ049L | Leptglossus zonatus | PQ197246 | SBEd | sv001 | 16S | pomegranate | AZ | tucson | UA | UAa8 | n/a |
| LZ050 | Caballeronia sp LZ050 | Leptglossus zonatus | GCA_031451105 | SBEa | sv013 | genome | pomegranate | AZ | tucson | MG | MGd9 | SNPsaurus^1^ |
| LZ052 | LZ052 | Leptglossus zonatus | PQ197247 | SBEa | sv012 | 16S | pomegranate | CA | bakersfield | WD | WDb9 | n/a |
| LZ053 | LZ053 | Leptglossus zonatus | PQ197248 | SBEa | sv016 | 16S | pomegranate | CA | fresno | RE | REd1 | n/a |
| LZ055 | LZ055 | Leptglossus zonatus | PQ197249 | SBEa | sv012 | 16S | pomegranate | CA | bakersfield | WD | WDb9 | n/a |
| LZ056 | LZ056 | Leptglossus zonatus | PQ197250 | SBEa | sv016 | 16S | pomegranate | CA | bakersfield | WD | WDa7 | n/a |
| LZ059 | LZ059 | Leptglossus zonatus | PQ197251 | SBEa | sv024 | 16S | pomegranate | CA | fresno | RE | REc1 | n/a |
| LZ060 | LZ060 | Leptglossus zonatus | PQ197252 | SBEa | sv002 | 16S | pomegranate | AZ | tucson | MV | MVc6 | n/a |
| LZ061 | LZ061 | Leptglossus zonatus | PQ197253 | SBEa | sv002 | 16S | pomegranate | AZ | tucson | MV | MVc6 | n/a |
| LZ062 | Caballeronia sp LZ062 | Leptglossus zonatus | GCA_031450785 | SBEa | sv013 | genome | pomegranate | AZ | tucson | MG | MGd9 | Plasmidsaurus^2^ |
| LZ063 | LZ063 | Leptglossus zonatus | PQ197254 | SBEa | sv013 | 16S | pomegranate | AZ | tucson | MG | MGd9 | n/a |
| LZ064 | LZ064 | Leptglossus zonatus | PQ197255 | SBEa | sv002 | 16S | pomegranate | AZ | tucson | MV | MVc6 | n/a |
| LZ065 | Caballeronia sp LZ065 | Leptglossus zonatus | GCA_031450975 | SBEa | sv016/sv241/sv444 | genome | pomegranate | CA | bakersfield | WR | WRa9 | SNPsaurus^1^ |
| LZ066 | LZ066 | Leptglossus zonatus | PQ197256 | SBEa | sv016 | 16S | pomegranate | CA | bakersfield | LG | LGd6 | n/a |
| SL2Y3 | Caballeronia sp SL2Y3 | Jalysus wickhami | GCA_022879575 | SBEa | sv024 | genome | Guara parviflora | AZ | tucson | Silverlake soccer park | see Stillson et al 2022 |  |

^1^ Sequenced on an Illumina HiSeq 4000 by SNPsaurus (Eugene, OR). Paired-end sequences were trimmed by bbduk (parameters: ktrim=r k=17 hdist=1 mink=8 ref=/bbmap/resources/nextera.fa.gz minlen=100 ow=t qtrim=r trimq=10 pigz=t unpigz=t), then assembled with SPAdes v3.13.0 using -k 77,99.

^2^ Sequenced by Plasmidsaurus (Eugene, OR) using Oxford Nanopore technology and assembled with Flye v. 2.9.
